# Synthesis, Processing, and Performance of a Furan-Based Glycidyl Amine Epoxy Resin

**DOI:** 10.1021/acsomega.5c05822

**Published:** 2025-11-13

**Authors:** Amy Honnig Bassett, Emre Kinaci, Giuseppe R. Palmese

**Affiliations:** Department of Chemical Engineering, Advanced Materials and Manufacturing Institute (AMMI), 3536 Rowan University, 201 Mullica Hill Rd., Glassboro, New Jersey 08028, United States

## Abstract

Furan diepoxy (FDE), a diglycidyl amine with a pendant furan, was synthesized using a 1:10 furfuryl amine/epichlorohydrin ratio. Aliquots were taken during the synthesis and characterized using gel permeation chromatography (GPC) and Fourier transform infrared spectroscopy (FTIR). Two significant products, FDE and α-chlorohydrin FDE, formed simultaneously with increasing reaction time. Eventually, α-chlorohydrin FDE became the main product. Purified FDE and α-chlorohydrin FDE were cured with an amine to investigate the network formation using differential scanning calorimetry (DSC), FTIR, and ^1^H and ^13^C­{^1^H} nuclear magnetic resonance (NMR). Dynamic mechanical analysis (DMA) was conducted to measure the thermomechanical properties and create a structure–property relationship. Compared to purified FDE, which had a storage modulus (*E*′) at 25 °C of 2.9 GPa and a *T*_g_ of 80 °C, α-chlorohydrin FDE had increased *E*′ at 25 °C of 4.4 GPa and *T*_g_ of 129 °C. The improved properties resulted from α-chlorohydrin FDE forming new cross-links via ring-opening of the furan.

## Graphical abstract

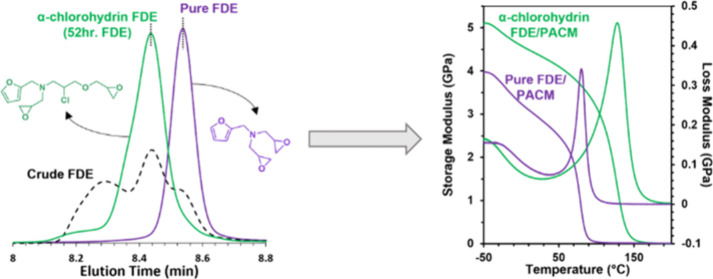

## Introduction

Epoxy resins are conventionally bisphenol A (BPA) based, with the diglycidyl ether of BPA (DGEBA) making up about 70% of the epoxy usage.^1,2^ BPA is a petroleum-based resource and a known human endocrine disruptor.^3,4^ With a growing focus on a more sustainable future, researchers have sought BPA alternatives. Literature has shown furan to be a promising candidate as a phenolic alternative because of its aromaticity and availability.^5,6^

Furan is a heterocyclic, five-membered ring extracted from polysaccharides found in biomass wastes.^5^ Furan-based compounds can possess a wide variety of functionality, including carboxyl, hydroxyl, amino, and vinyl moieties, leading to ease of use in multiple chemistries.^6^ Epoxy-amine chemistry using furan-based compounds has been explored in numerous studies. Hu et al. successfully synthesized the furan-based epoxy monomer, 2,5-bis­[(2-oxiranylmethoxy)-methyl]-furan (BOF) from 2,5-bis­(hydroxymethyl) furan (BHMF). BOF cured with conventional aliphatic and aromatic amines had an increased room temperature storage modulus (E’) compared to benzene-based analogs. The improved property was hypothesized to result from potential hydrogen bonding associated with the oxygen atom in the furan ring.^7^ A fully furan-based epoxy-amine system was also synthesized and cured. BOF was cured with difuran diamine (DFDA), synthesized from furfuryl amine. BOF/DFDA had a further increased *T*_g_ and room temperature E’ compared to BOF cured with a conventional aliphatic amine. The reasoning was again attributed to the potential hydrogen bonding of the oxygen in the furan ring with hydroxyl groups resulting from the epoxy-amine ring opening reaction. Additionally, the fully furan-based system also had increased thermal stability with a char yield in nitrogen at 750 °C of 40 wt %.^8^ Other investigators found similar results when using 2,5-furan dicarboxylic acid (FDCA) as the starting furan material. The epoxy monomer based on FDCA cured with 3, 3′-diamino diphenyl-sulfone (33DDS) and 4, 4′-diamino diphenyl-sulfone (44DDS) had excellent E’ and *T*_g_ compared to nonfuran-based systems.^9^ Potential hydrogen bonding and increased chain packing were attributed to the improved properties. The researchers proposed similar ideas for why furan increased viscoelastic properties, but did not provide fundamental evidence. The above-mentioned furan-based molecules require relatively complicated synthesis and purification processes to obtain thermosetting materials and have limited commercial availability. However, some furan-based molecules, such as furfuryl amine, are commercially available and can be readily functionalized to create thermoset materials. This investigation seeks to fundamentally understand the influence of the furan ring on (1) epoxy monomer synthesis, (2) epoxy-amine thermoset processing, and (3) polymer performance characteristics. The outcomes of this work will provide evidence for the role furan plays in improving the viscoelastic properties and promote the use of furan in thermosetting systems.

Epoxidizing furfuryl amine creates glycidyl amines instead of the glycidyl ethers shown in literature with BPA-based systems. Glycidyl ethers are characterized by an oxygen atom connected to the oxirane group and are the most widely used epoxy resins. Due to their popularity, extensive research has been done to fundamentally understand the synthesis and curing procedures to obtain the best properties with these materials.^10,11^ Glycidyl amines, however, are less common, leading to a more limited fundamental understanding of these systems for achieving the best properties. Glycidyl amines are characterized by a nitrogen atom connected to the oxirane group. Generally, epoxies are synthesized using epichlorohydrin and an acidic hydroxyl group for glycidyl ethers or an amine-containing compound for glycidyl amines.^11,12^ Despite similar synthetic routes, glycidyl amines present a unique challenge because of the formation of a tertiary amine upon complete glycidation. Tertiary amines are known to catalyze an epoxy’s ring opening, leading to homopolymerization and potentially creating unwanted side products during synthesis or cure.^10,12^

Limited foundational work has been done on glycidyl amine synthesis or cure. In the 1990s, a series of papers was published analyzing the impurities formed during the synthesis of glycidyl amines, the kinetics of intramolecular cyclization, and shelf life analysis.^13–17^ A review was also published focused on the kinetics and mechanisms of cure of glycidyl amines.^18^ Since the publication of these papers, there has been limited research on glycidyl amines. Additionally, the published papers focused on aniline-based molecules. Incorporating furan into a glycidyl amine will introduce further complications because of the structural differences between furan and benzene rings. To the best of our knowledge, there is no published work on understanding the interaction of the furan ring during the synthesis and cure of glycidyl amine.

This work has three aims: (1) to investigate the effect of synthetic conditions on glycidyl amine monomer formation, (2) to determine the interaction of the furan ring with glycidyl amine products during polymerization, and (3) to evaluate how the synthetic and polymerization conditions impact the final polymer properties of a furan-based glycidyl amine. The outcomes of this work will provide evidence for the unique ability of the furan ring to interact with synthetic products that are specific to glycidyl amine chemistry and how this can be tailored to impact the final polymer properties.

## Experimental Section

### Materials

Furfuryl amine (≥99%), furfuryl alcohol (98%), hydrochloric acid (37% aqueous solution), potassium hydroxide pellets (≥85%), toluene (≥99.5%), and methacrylic acid (99%) were purchased from Sigma-Aldrich. (±)-epichlorohydrin (99%), ethyl acetate (≥99.5%), hexane (mixture of isomers, ≥ 98.5%), sodium chloride (≥99.0%), magnesium sulfate heptahydrate, sodium hydroxide 50% (w/w) in aqueous solution, furfuryl glycidyl ether (97%), chloroform-d (99.8 atom % D + 0.03% (v/v) TMS stabilized), and tetrahydrofuran were purchased from VWR. AMC-2 catalyst was purchased from AMPAC Fine Chemicals. Amicure PACM curing agent (bis­(*p*-aminocyclohexyl) methane) was purchased from Evonik. All chemicals were used without further purification.

### Overview of the Four FDE Resins Synthesized and Evaluated

The synthesis of FDE was developed based on the well-studied glycidyl ether reactions. Reaction conditions, including epichlorohydrin concentration and reaction time, were varied to synthesize four FDE resins.

The first reaction was designed to utilize the ideal molar concentration of 1:2 furfuryl amine: epichlorohydrin, which resulted in the first FDE resin named S-FDE. S-FDE contained the desired FDE monomer in addition to high molecular weight oligomers. The second reaction was designed to minimize the high molecular weight oligomers, and an increased molar concentration of 1:10 furfuryl amine: epichlorohydrin was used with a 24 h reaction time for adduct formation. The resulting resin was considered the second resin and named 1:10-FDE. 1:10-FDE had minimal high molecular weight oligomers but contained a mixture of desired FDE and a side product. The formation of the side product was investigated in the third reaction by varying the epoxy adduct formation time, and aliquots were taken between 2 and 52 h. After 52 h, the resulting resin no longer contained the desired FDE but primarily the side product from 1:10-FDE. The side product was determined to be α-chlorohydrin FDE and was referred to as 52 h.-FDE, which is the third resin. The last FDE resin was prepared using flash liquid chromatography to obtain a pure FDE monomer, which was labeled Flashed-FDE.

### Synthesis for Stoichiometric FDE (S-FDE)

A molar ratio of 1:2.1 furfuryl amine: epichlorohydrin was used to synthesize S-FDE. Furfuryl amine (38.8 g, 0.40 mol) and epichlorohydrin (77.6 g, 0.84 mol) were added to a 500 mL 4-necked round-bottom flask equipped with a constant pressure dropping funnel, overhead mixer, thermometer, and condenser. A phase transfer catalyst was not needed because of the compatibility of furfuryl amine and epichlorohydrin at room temperature. The mixture was reacted with continuous stirring for 24 h at 10–15 °C in an ice bath. The reaction was cooled in an ice bath to 0 °C before adding 50% (w/w) sodium hydroxide dropwise (64.0 g, 1.6 mol). The reaction mixture was allowed to react for another 2 h before being dissolved in 500 g of ethyl acetate and washed with 500 g of brine two times. The organic layer was collected after each wash. After the final brine wash, the organic layer was dried over magnesium sulfate. The reaction mixture was purified using two methods. The first method used vacuum distillation to remove ethyl acetate and unreacted epichlorohydrin. The final product was a light-yellow, low-viscosity liquid named S-FDE, which contained reaction products of various molecular weights along with the desired FDE monomer. The second method used flash liquid chromatography to purify the distilled reaction products further to isolate pure FDE. Ethyl acetate/hexanes were used for this purpose. The final product was also a light-yellow, low-viscosity liquid named Flashed-FDE.

### Synthesis for 1:10-FDE Aliquots

A similar synthesis procedure was followed as above, except with excess epichlorohydrin in a 1:10 furfuryl amine: epichlorohydrin molar ratio to help eliminate the generation of high molecular weight oligomers. Furfuryl amine (174.8 g, 1.8 mol) and epichlorohydrin (1665.4 g, 18 mol) were added to a 3000 mL 4-necked, round-bottom flask equipped with an overhead mixer, thermometer, and condenser and reacted with continuous stirring at 10–15 °C in an ice bath. Equal aliquots of 204.5 g were taken after 2 h, every 4 h from 4 to 12, 24, 31, and 52 h. After the allotted reaction time, each aliquot was cooled to 0 °C in an ice bath, and 50% (w/w) sodium hydroxide (32.0 g, 0.80 mol) was added dropwise and allowed to react for 2 h. Each aliquot was dissolved in ethyl acetate and washed with brine two times. The organic layer was collected after each wash and dried over magnesium sulfate after the final wash. Vacuum distillation was used to remove ethyl acetate and unreacted epichlorohydrin. The low-time products (2 and 4 h) were dark brown and high-viscosity liquids with low yields (1% for FDE). Starting at 8 h up to 52 h, the products were light yellow, low-viscosity liquids with FDE yields of 70–75%. The yields are low due to the resins not forming much FDE, but rather oligomers and chlorinated compounds. The resulting resins were named by their reaction time, for example the system reacted for 8 h before ring closing with sodium hydroxide is called 8 h.-FDE. Note that 24 h.-FDE is also referred to as 1:10-FDE

### Synthesis of Furfuryl Glycidyl Ether (FGE)

Furfuryl glycidyl ether (FGE) was also synthesized using a method similar to 1:10-FDE to investigate the reaction differences between glycidyl ethers and glycidyl amines. Synthesis details are found in the Supporting Information (SI).

### Resin Characterization

All FDE and FGE resins were characterized using gel permeation chromatography (GPC). A Waters AQUITY Advanced Polymer Chromatography unit with an AQUITY refractive index detector was used. Tetrahydrofuran (THF) Optima was used as the eluent with a flow rate of 0.6 mL min^–1^. A series of 4.6 × 150 mm AQUITY APC columns (XT 450 2.5 μm, XT 125 2.5 μm, and XT 45 1.7 μm) was used and maintained at 40 °C. Polystyrene standards were used to calibrate the instrument (PSS ReadyCal Kit, range: 474–2,500,000 g mol^–1^; maximum Đ: 1.15). Samples were prepared by mixing 10–12 mg of resin into 2 mL of THF. The number-average molecular weight (*M*_n_) and areas under the curve were determined. The hydrolyzable chlorine content was measured for Flashed-FDE and 52 h.-FDE following ASTM D1726–11 using Test Method A.^19^ Test method A is used for 1 wt % or less hydrolyzable chlorine content.

The epoxy equivalent weight (EEW) was measured for Flashed-FDE and 52 h.-FDE. The EEW was measured following ASTM D1652–19 using the manual titration method.^20^ However, it was found that for monosubstituted furan epoxies, the furan ring interacted with the perchloric acid used in the titration. A new titration method was developed to obtain an accurate EEW for monosubstituted furan epoxies based on methacrylation and acid number titration. Complete details on the EEW method are found in the SI.

Samples were also characterized by ^1^H- and ^13^C­{^1^H}-NMR (24 °C in CDCl_3_) using a Bruker Avance Neo 400 MHz NMR Spectrometer. ^1^H NMR spectra with peak assignments are found in Figures S2–S4. Full ^13^C­{^1^H}-NMR spectra are found in Figures S5–S7. The ^1^H NMR chemical shifts, number of protons represented by the signal, peak multiplicity, and coupling constants are listed below for the two pure products of Flashed-FDE and 52 h.-FDE. Additionally, the ^13^C­{^1^H}-NMR chemical shifts are denoted below for Flashed-FDE and 52 h.-FDE.

Flashed-FDE: ^1^H NMR (24 °C in CDCl_3_, 400 MHz): δ_H_ 7.38 (s, 2H), 6.32 (s, 2H), 6.24 (s, 2H), 3.96 (d, J= 14.6, 1H), 3.86 (s, 2H), 3.7 (d, J = 15.1, 1H), 3.11 (m, 4H), 3.04 (dd, J= 13.9, 3.2, 2H), 2.90 (dd, J= 13.9, 3.7, 2H), 2.76 (t, J= 4.2 (x2), 4H), 2.62 (dd, J= 14.2, 6.3, 2H), 2.51 (ddd, J= 10.4, 5.0, 2.7, 4H), 2.38 (dd, J = 13.7, 6.8 2H). ^13^C­{^1^H}-NMR (24 °C in CDCl_3_, 100 MHz): δ_C_ 151.8 (d, J = 13.8). 142.2 (d, J = 4.4), 110.1 (d, J= 1.5), 10.9.2 (s), 109.0 (s), 56.8 (s), 56.2 (s), 51.2 (d, J = 3.6), 50.5 (s), 45.1(s), 44.7 (s).

52 h.-FDE: ^1^H NMR (24 °C in CDCl_3_, 400 MHz): δ_H_ 7.39 (s, 2H), 6.33 (s, 2H), 6.23 (s, 2H), 3.88 (m, 2H), 3.82 (dd, J = 5.7, 4.1, 2H), 3.77 (m, 2H), 3.56 (m, 2H), 3.47 (d, J = 17.6, 2H), 3.12 (m, 2H), 3.05 (m, 2H), 3.01 (d, J = 2.8, 2H), 2.97 (d, J = 2.9, 2H), 2.92 (m, 2H), 2.89 (m, 2H), 2.83 (d, J= 4.1, 2H), 2.77 (m, 2H), 2.72 (d, J = 6.5, 2H), 2.63 (m, 2H), 2.54 (m, 2H), 2.38 (m, 2H). ^13^C­{^1^H}-NMR (24 °C in CDCl_3_, 100 MHz): δ_C_ 151.8 (d, J = 2.9). 142.4 (s), 110.2 (d, J= 1.5), 109.2 (d, J = 2.9), 69.1 (s), 68.7 (s), 68.2 (s), 67.9 (s), 58.2 (s), 57.8 (s), 57.5 (s), 57.2 (s), 56.8 (m), 56.2 (s), 56.1 (s), 51.6 (s), 51.5 (s), 51.3 (s), 51.2 (s), 50.9 (s), 50.7 (s), 50.5 (s), 47.3 (d, J = 8.7), 47.0 (d, J= 13.8), 45.2 (s), 45.1 (s), 44.8 (s), 44.6 (s).

### Reaction Kinetics for S-FDE and 1:10-FDE

A Thermo Scientific Nicolet iS50 FTIR spectrometer was used in transmission mode in the near-IR range (4000–8000 cm^–1^) to track the formation of the epoxy-amine adduct over time. The reaction mixtures of 1:2.1 and 1:10 furfuryl amine: epichlorohydrin were mixed at room temperature and put into the FTIR cell setup. The cell setup was a sealed 1.5 mm outer diameter glass tube. A spectrum was taken every 30 min for 52 h, and each spectrum was 32 scans with a 4 cm^–1^ resolution. The peak height of 5987 cm^–1^ was used as a reference. The conversion of epoxy (EP, 6080 cm^–1^), secondary amine (SA, 6545 cm^–1^), and primary amine (PA, 4945 cm^–1^) were calculated using eqs 1–3, where I­(t = 0) and I­(t) are the designated peak intensities before and during cure:^21–23^

$$Conversion(\alpha_{EP},\%)=1-\left[\frac{\frac{I(t{)}_{6080\;cm^{-1}}}{I(t{)}_{5987\;cm^{-1}}}}{I(t=0{)}_{6080\;cm^{-1}}}\right]$$ 1

$$Conversion(\alpha_{PA},\%)=1-\left[\frac{\frac{I(t{)}_{4945\;cm^{-1}}}{I(t{)}_{5987\;cm^{-1}}}}{I(t=0{)}_{4945\;cm^{-1}}}\right]$$ 2

$$Conversion(\alpha_{SA},\%)=1-\left[\frac{\frac{I(t{)}_{6545\;cm^{-1}}}{I(t{)}_{5987\;cm^{-1}}}}{I(t=0{)}_{6545\;cm^{-1}}}\right]$$ 3

### Curing Mechanism of FDE Resins

Differential scanning calorimetry (DSC) was used to determine the curing temperature and *T*_g_ of Flashed-FDE and 52 h.-FDE cured with PACM at measured stoichiometries. A TA Instruments Discovery DSC 2500 was used. Approximately 10 mg of the sample was placed into a Tzero pan with a Tzero Hermetic lid. The samples were heated from 0 to 250 °C at 1 °C min^–1^, cooled to 0 °C before heating again from 0 to 250 °C with a ramp of 2 °C min^–1^ in a nitrogen atmosphere (flow of 50 mL min^–1^ and balance of 300 mL min^–1^). The curing temperature was determined as the peak of the exotherm, and *T*_g_ was defined as the inflection point on the second ramp. Table S3 shows the peak enthalpies of Flashed-FDE/PACM and 52 h.-FDE/PACM cured with the stoichiometric ratio of 1:2 amine: epoxy and Table S4 shows the peak enthalpies of these systems using 10% less amine than the stoichiometric ratio of 1:2 amine: epoxy.

The 52 h.-FDE sample was further studied to investigate the effect of molecular structure on network formation. In order to control network formation, 52 h.-FDE was cured with furfuryl amine in excess of 10 wt %. A Thermo Scientific Nicolet iS50 FTIR spectrometer was used in transmission mode. A spectrum was taken before and after the cure at 160 °C for 12 h. Each spectrum was 32 scans with a 4 cm^–1^ resolution. A Bruker Avance Neo 400 MHz NMR Spectrometer was used to collect ^1^H- and ^13^C­{^1^H}-NMR (24 °C in CDCl_3_) at 25 °C, 60 °C for 2 h, 120 °C for 2 h, and 160 °C for 2 h.

### Curing of FDE Resins for Polymer Properties

All FDE resins were cured with PACM assuming a theoretical EEW of Flashed-FDE (1:2 amine: epoxy by weight). Aliquots from 8 to 52 h were cured because FDE in those samples allowed cross-linking. Silicon rubber molds with uniform dimensions were used to cure the samples. Each resin was degassed before pouring the resin into the molds. The resin was cured at 60 °C for 2 h, 120 °C for 2 h, and 160 °C for 3 h. The cured samples were cooled to room temperature before being removed from the molds. The resulting polymers were labeled Flashed-FDE/PACM, S-FDE/PACM, and for the aliquots as the reaction time/PACM (e.g., 8 h.-FDE/PACM). The mass of the resin was measured before cure, and the mass of the polymer was measured after cure. The mass difference was determined as the mass loss during cure, and the results are found in Table S5.

### Polymer Properties

A TA Instruments Discovery DMA 850 was used to determine the viscoelastic properties of all cured FDE samples. Samples were prepared with appropriate dimensions of 35 × 12 × 2.5 mm^3^. An oscillatory deflection amplitude of 10 μm, a frequency of 1.0 Hz, and a Poisson’s ratio of 0.35 were used. Samples were heated from −50 to 200 °C at 2 °C min^–1^. Each sample was run twice, and the second run was used for the final reported properties. The storage modulus (E′) at room temperature was recorded, and the maximum temperature of the loss modulus (E″) was used as a measure of *T*_g_. Tan δ peak temperatures were also recorded; the results are found in Figure S11. The rubbery modulus was measured at a temperature of *T*_g_ plus 30 °C. The cross-linking density (*v*_*e*_) was calculated using eq 4, where R is the ideal gas constant (8.314 cm^3^ MPa K^–1^ mol^–1^), T is the temperature in kelvin of *T*_g_+30 °C, and E′ is the rubbery modulus at *T*_g_+30 °C^24^

$$v_{e}=\frac{E^{’}}{3RT}$$ 4

The density of the cured polymer samples at 25 °C was measured using a density gradient column following ASTM D1505–18.^25^ Samples were cut into cubes of 3 mm^3^ and were submerged in deionized (DI) water at 25 °C for 10 min. After 10 min, the samples were introduced into the density gradient column. The densities were determined using the graphical calculation method. The average of three samples was taken as the density.

## Results and Discussion

### Investigating the Effect of Reaction Conditions on FDE Resin Synthesis

The reaction scheme in Figure 1 shows how furfuryl amine was epoxidized using epichlorohydrin. A slight excess of epichlorohydrin (2.1 mol) was used to minimize oligomer formation.^11^ Furfuryl amine and epichlorohydrin are soluble at room temperature, eliminating the need for a phase transfer catalyst, which is commonly used in the synthesis of DGEBA.^12^ Two purification methods were used to remove unreacted epichlorohydrin. The first method used vacuum distillation and the second used flash chromatography.

**Figure 1:**
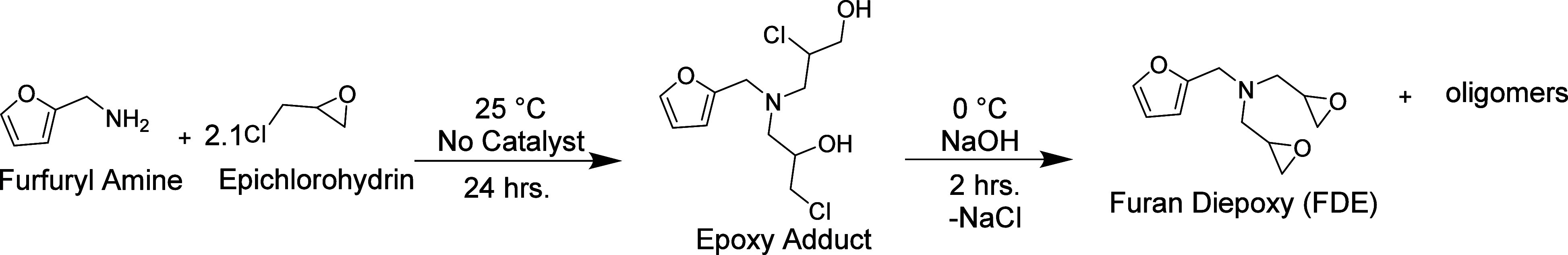
Reaction scheme for FDE.

Figure 2 includes the GPC traces for S-FDE and Flashed-FDE. The S-FDE trace had three peaks, suggesting the presence of high-molecular-weight oligomers in addition to pure FDE. Comparing the GPC traces of S-FDE and Flashed-FDE shows the location of the pure FDE peak, as Flashed-FDE has one peak at 8.56 min with an *M*_n_ value of 209 Da based on polystyrene standards. The molecular weight of FDE is 210 Da. Flash chromatography successfully removed oligomers and unreacted epichlorohydrin.

**Figure 2:**
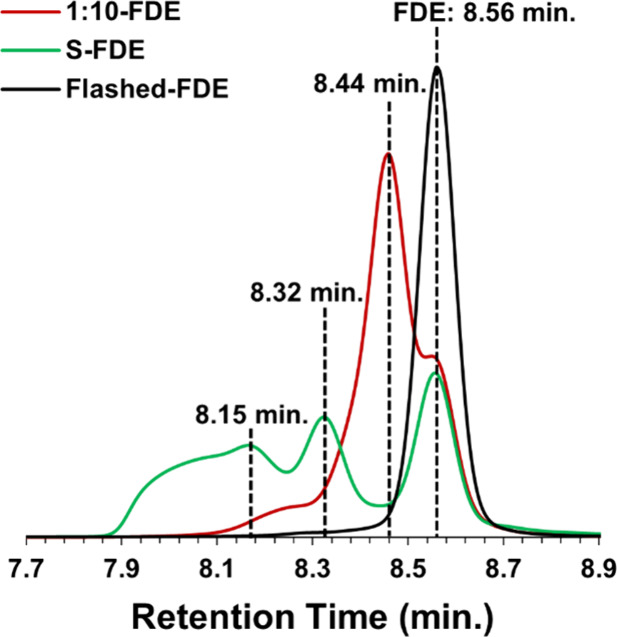
GPC trace for S-FDE, 1:10-FDE, and Flashed-FDE

The GPC peak areas were used to calculate the percentage of pure FDE formed in S-FDE. By this method, S-FDE contained about 44% pure FDE, with the remaining being high-molecular-weight oligomers.

Using excess epichlorohydrin well above the stoichiometric value in the epoxidation of bisphenols and other alcohols yields products with limited oligomerization wherein the amount of *n* = 0 monomers can be more than 85%.^12^ In an attempt to eliminate both the oligomers formed and the need for flash chromatography to purify the epoxidation products of furfuryl amine, a higher molar ratio of furfuryl amine to epichlorohydrin (1:10 mol) was used. Figure 2 also shows the GPC trace for the epoxidation products of 1:10-FDE.

The GPC of 1:10-FDE has three peaks corresponding to low molecular weight products. As expected, increasing epichlorohydrin concentration eliminated the oligomers observed in S-FDE. However, in addition to a side shoulder at 8.56 min corresponding to FDE, a new peak at 8.44 min, which was not observed in S-FDE, became the primary product. Based on peak area analysis, about 18% of the product is FDE, and the new product formed comprises 78% of the mixture. Thus, epoxidizing furfuryl amine using a 1:10 molar ratio of furfuryl amine to epichlorohydrin created an unexpected product and minimized FDE formation. To better understand this synthetic process, the influence of time on the furfuryl amine-epichlorohydrin adduct formation and its effect on the resultant epoxy products was investigated.

For a 1:10 molar ratio of furfuryl amine to epichlorohydrin reaction, aliquots were taken at intervals during a reaction period of 2–52 h. Each aliquot was treated with sodium hydroxide, purified using vacuum distillation, and analyzed using GPC. Figure 3 shows GPC traces of the resulting products with increasing reaction time. Table S2 provides a detailed table of elution times and calculated concentrations for each aliquot. At low reaction times of 2 and 4 h, broad peaks with low elution times (7.7–8.1 min) were present. The peaks in both samples were mainly high molecular weight oligomers that resulted from unreacted furfuryl amine and epichlorohydrin reacting during vacuum distillation up to 80 °C. FDE was not formed in detectable quantities because the epoxy adduct did not form completely.

**Figure 3:**
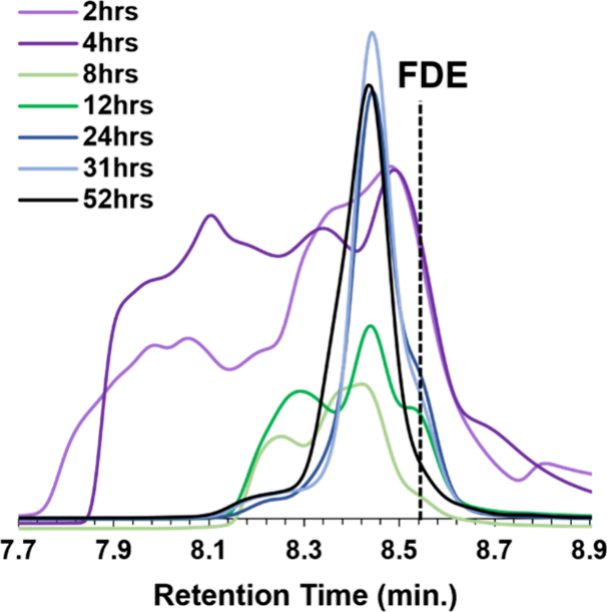
GPC traces for purified 1:10-FDE with increasing reaction time from 2 to 52 h.

Epoxies obtained from adducts produced by 8 h of reaction yielded products that did not show the presence of high molecular weight oligomers. Two peaks and a shoulder were observed. The two peaks were at 8.25 and 8.44 min, with a third peak appearing at 8.56 min. From the previous GPC data, the peak at 8.56 min is attributed to FDE, and the peak at 8.44 min corresponds to the unexpected primary product. The peak at 8.25 min results from lower molecular weight oligomers than those observed for the 2 and 4 h adducts.

An adduct reaction time of 12 h produces GPC traces with three well-resolved peaks. However, as the adduct reaction time proceeded beyond 12 h, the unexpected peak at 8.44 min became dominant. By 52 h, both the FDE peak and the low molecular weight oligomer peak are reduced to the point where a single peak at 8.44 min is observed.

Based on peak areas, 12 h adducts resulted in a maximum FDE concentration of about 21% in the product. With increasing adduct reaction time, the percentage of FDE obtained decreases, eventually reaching undetectable levels in the case of a 52 h reaction time. In parallel, the unexpected major peak (8.44 min) was found to increase with increasing adduct reaction time until it appeared to be the only product formed in the case of a 52 h adduct reaction. Using excess epichlorohydrin for adduct formation results in the FDE precursor being an intermediate product, suggesting a more significant second reaction is occurring compared to the primary reaction. This second reaction must occur between the epoxy adduct and the excess epichlorohydrin. A likely possibility is that the hydroxyl resulting from adduct formation reacts with epichlorohydrin when given enough time to create a chlorinated product after the ring closes, resulting in hydrolyzable chlorine. A similar reaction, though minor, has been observed during the epoxidation of BPA.^12^ However, this secondary reaction could be significantly faster in glycidyl amines, potentially occurring to high extents during adduct formation.

For the epoxidation scheme shown in Figure 1, the first step can be monitored using Near-IR to provide insight into how increasing the epichlorohydrin concentration and changing adduct reaction time creates a distribution of products. Near-IR was used to track the primary/secondary amine functionality on furfuryl amine and the epoxy peaks on epichlorohydrin as they reacted at room temperature with a reaction time of 52 h. The Near-IR spectra for 1:10-FDE are shown in Figure S1, in which the primary amine (4945 cm^–1^), secondary amine (combination peak with primary amine, 6545 cm^–1^), and epoxy peaks (4530 and 6080 cm^–1^) are labeled. Because of the overlapping peak from furfuryl amine in the main epoxy peak (4530 cm^–1^),^21–23^ the epoxy overtone (6080 cm^–1^) peak was used to measure epoxy conversion. eqs 1–3 were used to quantify the conversions of epoxy and amine moieties. Figure 4 shows the conversion values calculated using eqs 1–3 of epoxy and amine moieties as a function of reaction time at room temperature for (A) 1:10-FDE and (B) S-FDE.

**Figure 4:**
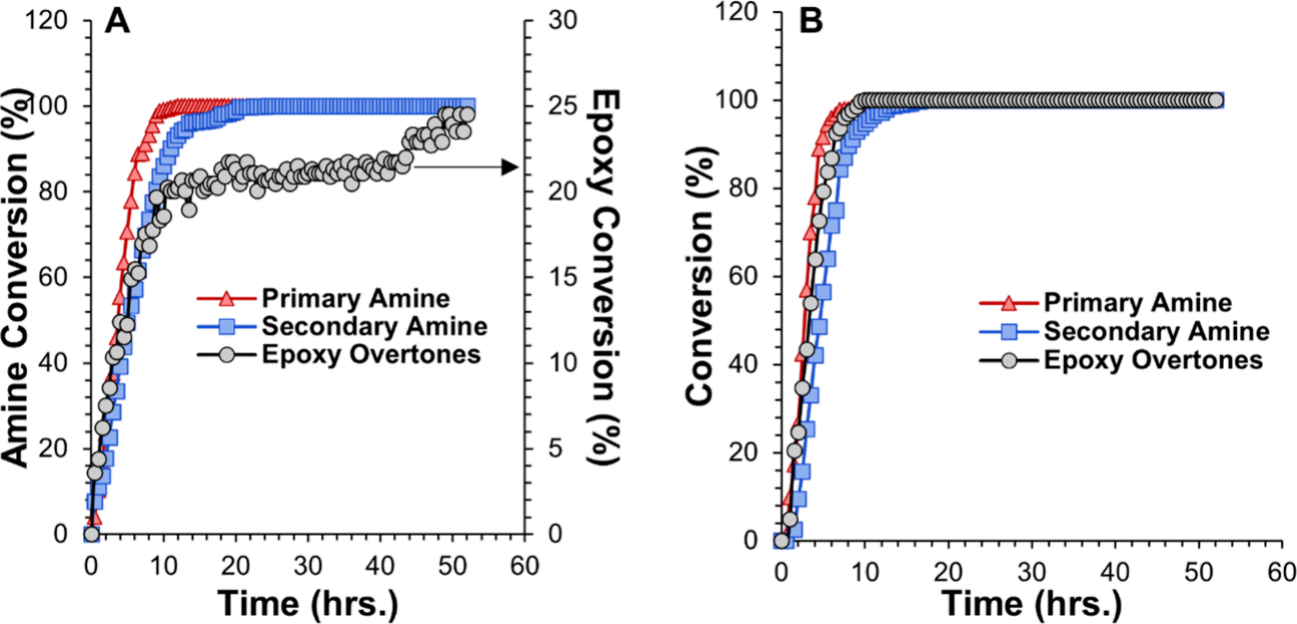
Primary amine, secondary amine, and epoxy kinetic curves using near-IR data. (A) 1:10-FDE (B) S-FDE.

For 1:10-FDE, the primary amines fully reacted after 10 h, and the secondary amines after 20.5 h. After 20.5 h of reaction, the epoxy conversion reaches a plateau of around 20%. However, continued reaction up to 52 h shows that the epoxy begins to react again, reaching a value of 25%. If epoxies only react with amines, then at most 20% of the epoxy groups would react after the secondary amines are consumed. The epoxy reacting after the secondary amines are consumed also suggests the presence of a second reaction consistent with the reaction of hydroxyls with excess epichlorohydrin.

The S-FDE conversion as a function of reaction time plot showed that the primary amines fully reacted after 10 h, and the epoxies reached complete conversion after the secondary amines were consumed. Unlike the 1:10-FDE, S-FDE had no excess epichlorohydrin. The lack of excess epichlorohydrin resulted in FDE and high molecular weight oligomers being the major products with no measurable unexpected side product.

^1^H NMR spectroscopy was used to characterize the products formed during synthesis. The 52-h product, though similar to Flashed-FDE, particularly with respect to the monosubstituted furan ring (Figures S2–S4), had noticeable differences in the epoxy hydrogen peaks and the hydrogens on the carbon bridge between the furan and the nitrogen. In both regions, there was peak broadening, suggesting the formation of a product containing additional chemical groups.

^13^C­{^1^H}-NMR spectroscopy was used instead to characterize the products formed during the synthesis because the spectra had more apparent differences. Figure 5 shows ^13^C NMR spectra (A) showing the furan ring carbon region, and (B) showing the carbons between the epoxy ring and furan. The full spectra are found in Figures S5–S7.

**Figure 5:**
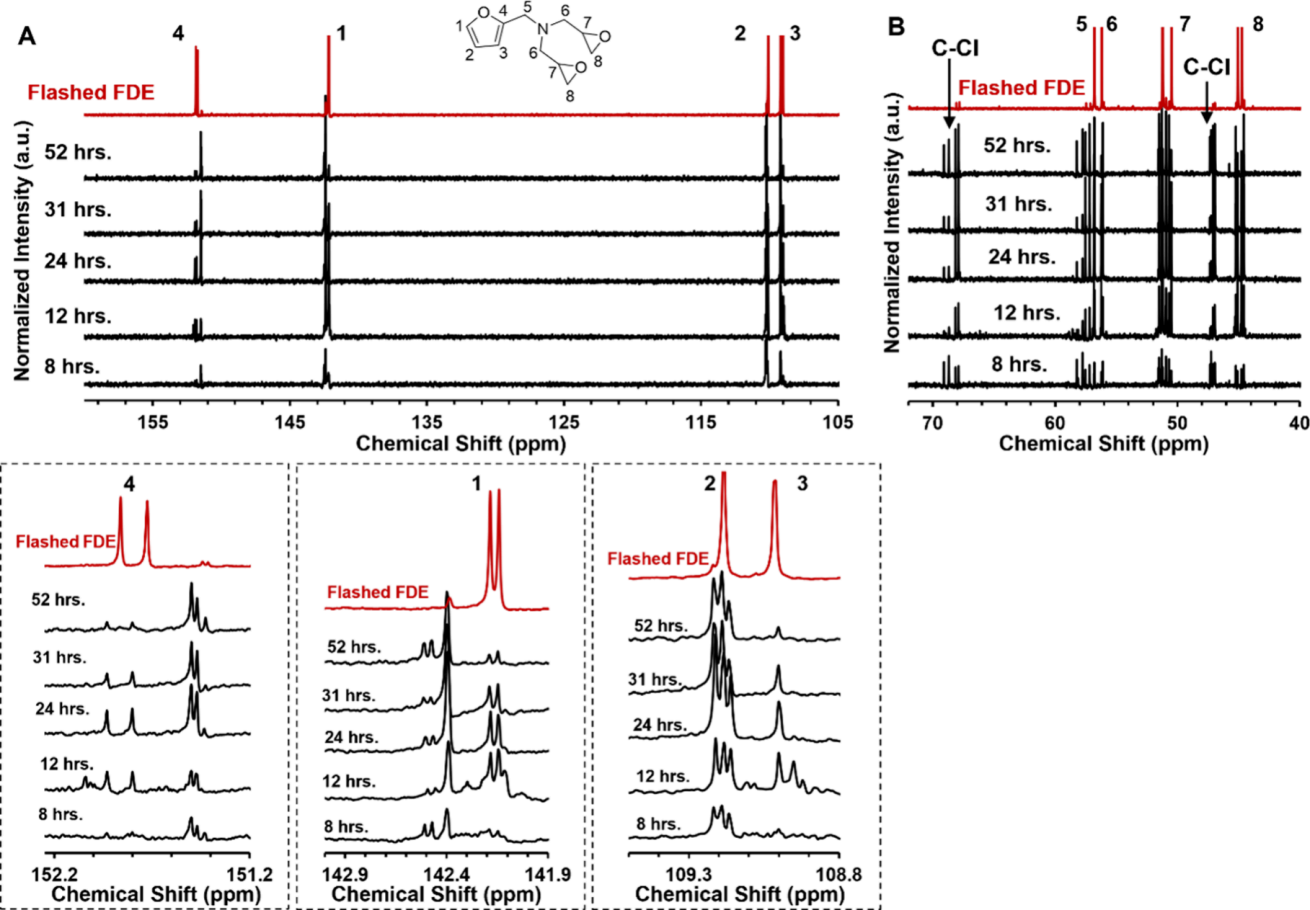
^13^C­{^1^H}-NMR for increasing reaction time of 1:10-FDE compared to Flashed-FDE with spectra offset for clarity. (A) Furan ring carbons with all carbons in the FDE structure labeled for all regions. Inserts are zoomed-in portions of the spectra. (B) Epoxy and chlorinated product carbons.

Flashed-FDE showed four peaks between 105 and 155 ppm for each of the carbons in the furan ring. The products from 8 to 52 h also had four peaks between 105 and 155 ppm, representing a pendant, monosubstituted furan ring present. However, different splitting patterns are observed when compared to Flashed-FDE, arising from structural differences.

The carbons on the two epoxy rings and bridge connecting the epoxy to the nitrogen split as three groups of doubles from 40 to 60 ppm for Flashed-FDE. The samples from 8 to 52 h had additional peaks between 46 and 48 ppm and 68–70 ppm that were not present in Flashed-FDE. This suggested that these peaks were associated with the carbons on the chlorinated product observed in GPC. Carbon-chlorine peaks are known to be found between 30 and 75 ppm, and the observed additional peaks fall within this range.^26^ ^13^C­{^1^H}-NMR results supported the hypothesis from the GPC findings of a chlorinated product forming that had a similar chemical structure to FDE.

Figure 6 shows the proposed reaction pathway for forming FDE and the chlorinated side product, α-chlorohydrin FDE, found in the 52 h product. Based on the GPC, only one hydroxyl was observed to react. If more than one hydroxyl reacted, it would have a shorter retention time in the GPC. The second hydroxyl did not respond appreciably, and the reason for this is still under investigation.

**Figure 6:**
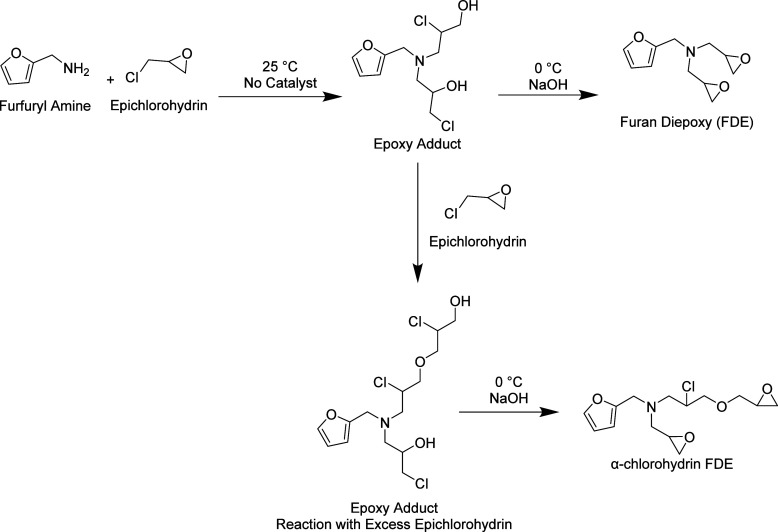
Proposed reaction mechanism for the formation of a chlorinated side product with a hydroxyl group in an open epoxy ring with excess epichlorohydrin.

### Resin Characterization

Hydrolyzable chlorine content was measured following Test Method A in ASTM D1726–19, a common measure of chlorine impurities.^19^ There was no clear trend with increasing reaction time. Each sample contained some chlorinated compounds, as was expected based on the GPC data. 8 h.-FDE had the highest percentage at 18 wt %, while the other reaction times showed a percentage between 11 and 13 wt %. Theoretically, the 52 h product contains 12 wt % chlorine. Flashed-FDE had less than 1 wt % hydrolyzable chlorine, confirming the removal of chlorinated side products using flash chromatography.

The GPC data showed that the chlorinated product (8.44 min) formed as early as 8 h, the same time FDE started forming. The hydrolyzable chlorine content supported the GPC and ^13^C­{^1^H}-NMR data. It also supported the parallel nature of FDE and chlorinated product formation. The hydrolyzable chlorine content confirmed that, on average, one chlorine atom was on the molecule based on the theoretical chlorine value.

EEW is an essential measure for characterizing epoxy moieties. EEW was measured using the standard titration method following ASTM D1652–11.^20^ Table 1 includes the values listed under “EEW-Standard titration.” Using the standard titration method, Flashed-FDE had an EEW of 77 g/eq The theoretical EEW for Flashed-FDE is 105 g/eq A lower-than-expected EEW could arise from excess epichlorohydrin, but the value would be closer to the molecular weight of epichlorohydrin (92 g mol^–1^). There is potential for the furan ring to participate in the titration, which was tested by titrating furfuryl alcohol. An EEW of the molecular weight (98 g/eq ) was determined, confirming the furan rings could react with the strong acids used for titration. The substitution of the furan ring is important for EEW titration because the literature showed that disubstituted furan rings could obtain accurate EEW values.^7^

**Table 1:** Epoxy Equivalent Weight (EEW) Using Standard Titration (ASTM D1652-19)^20^ and a Newly Developed Method Based on Acid Number Titration Compared with the Theoretical EEW Values

system	EEW-standard titration (g/eq)	EEW-acid number titration (g/eq)	theoretical EEW (g/eq)
52 h.-FDE		160	151
Flashed-FDE	77	113	105
EPON828	188	180	186

A new titration method was developed to more accurately measure the EEW of monosubstituted furan epoxy resins based on methacrylation and acid number titration.^27^ The EEW value for Flashed-FDE was calculated to be 113 g/eq and the theoretical value was 105 g/eq There was good agreement between the two values. The method was confirmed using Epon828, the diglycidyl ether of bisphenol A, which had an EEW of 186 g/eq as provided by the supplier. Using the acid number titration method, Epon828 had an EEW of 180 g/eq With good agreement between the measured and theoretical values for Flashed-FDE and Epon828, the EEW was determined for 52 h.-FDE. Theoretically, the EEW of 52 h.-FDE is 151 g/eq After methacrylating 52 h.-FDE, the calculated EEW was 160 g/eq The good agreement between the theoretical and calculated values further confirmed the structure of 52 h.-FDE to be α-chlorohydrin FDE. The EEW was not measured for the other samples in the time study because the main interest was in the formation of the chlorinated product.

### Synthesis of Furfuryl Glycidyl Ether (FGE) to Compare Glycidyl Ether Chemistry to Glycidyl Amine Chemistry

The high reactivity of amines with epoxies creates a unique chemistry during the epoxidation reaction. To confirm the effect the tertiary amine has on the glycidation of an amine, furfuryl alcohol was chosen as a furan hydroxyl analog to furfuryl amine, creating furfuryl glycidyl ether (FGE). Excess epichlorohydrin was used to epoxidize furfuryl alcohol in the same ratio as 1:10-FDE and left to react for 52 h. The reaction was purified using the same method as 1:10-FDE, and GPC was used to confirm the product formation. FGE did not form a chlorinated product, unlike FDE, as shown by the GPC trace in Figure S8. The glycidation of an amine-containing compound results in a unique chemistry, where the presence of tertiary amines promotes the formation of a chlorinated epoxy product, whereas this phenomenon was not observed in the glycidation of a hydroxyl-containing compound.

Overall, the synthesis of glycidyl amines varies from the well-studied synthesis of glycidyl ethers. Due to the difference in chemical nature between an amine and a hydroxyl, glycidyl amines have the potential to form a chlorinated product, 52 h.-FDE or α-chlorohydrin FDE, when using excess epichlorohydrin. The following section will investigate the influence of the molecular structure on epoxy-amine polymerization by focusing on Flashed-FDE and 52 h.-FDE. The significant difference between Flashed-FDE and 52 h.-FDE is the internal chlorine atom on 52 h.-FDE.

### Curing of Flashed-FDE and 52 h.-FDE with PACM

Flashed-FDE and 52 h.-FDE were cured with PACM to create cross-linked networks. Flashed-FDE was compared to 52 h.-FDE because it was the purest product and did not contain α-chlorohydrin FDE. DSC was used to investigate the curing behavior. Samples were heated at 1 °C min^–1^ from 0 to 250 °C on a first ramp to measure the curing exotherm and had a second ramp of 2 °C min^–1^ from 0 to 250 °C to measure the *T*_g_. Figure 7 shows the DSC thermograms for both epoxies cured with PACM with A) the first heating ramp for the exothermic curing peak and B) the second heating ramp for *T*_g_ as well as the reaction schemes for C) Flashed-FDE cured with PACM and D) 52 h.-FDE cured with PACM. Table 2 presents the peak temperatures of the first ramp, *T*_g_ values as the inflection point found with the second heating, and the EEW value used to calculate stoichiometric mixtures.

**Table 2:** DSC Values for Flashed-FDE and 52 h.-FDE Cured with PACM to Determine Curing Temperature and *T*_g_

system	peak temp. 1 (°C)	peak temp. 2 (°C)	peak temp. 3 (°C)	*T*_g_ (°C)	EEW (g/eq.)
Flashed-FDE/PACM	103			79	105
52 h.-FDE/PACM	68	139	194	177	208

**Figure 7:**
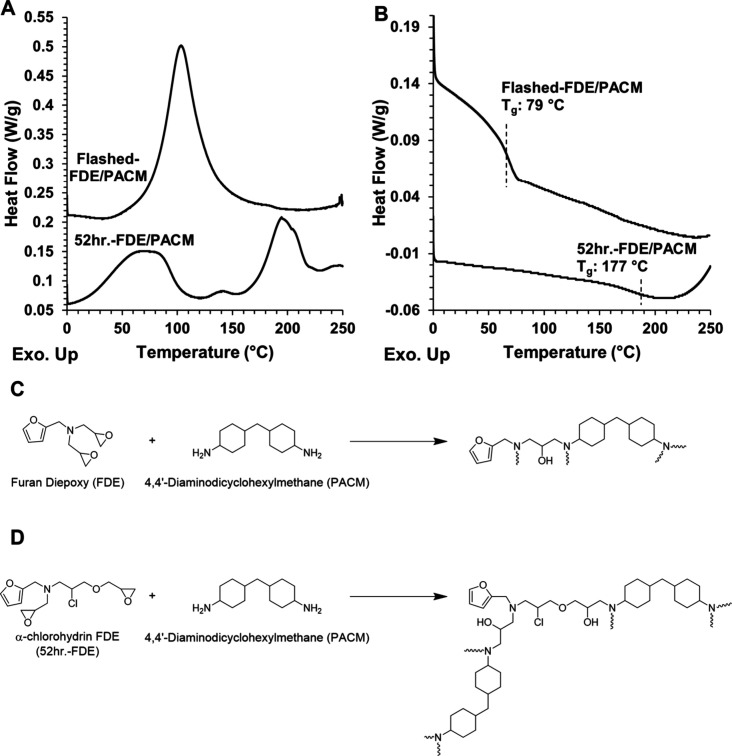
A) DSC thermogram for first heating ramp from 0 to 250 °C heating at a rate of 1 °C min^–1^ to determine exothermic curing peaks. B) DSC thermogram for second heating ramp from 0 to 250 °C heating at a rate of 2 °C min^–1^ to determine T_g._ C) Reaction scheme for Flashed-FDE polymerized with PACM. D) Reaction scheme for 52 h.-FDE polymerized with PACM.

For Flashed-FDE/PACM, a single exothermic peak was observed at a peak temperature of 100 °C, which was attributed to the epoxy-amine reaction. In contrast, 52 h.-FDE/PACM exhibited three distinct exothermic peaks, indicating the presence of additional reactions. The first peak, similar to Flashed-FDE/PACM, resulted from the epoxy-amine reaction but peaked at a lower temperature of 68 °C. Notably, the onset was earlier in 52 h.-FDE/PACM than Flashed-FDE/PACM. For Flashed-FDE/PACM, the peak started forming near 40 °C, whereas for 52 h.-FDE/PACM, the onset temperature was between 15 and 20 °C. These lower onset and peak temperatures of the epoxy-amine reaction for 52 h.-FDE/PACM were attributed to the chlorine, which is known to accelerate the reaction due to the polar carbon-chlorine bond. The chlorine is considered an electron withdrawing group and will lower the electron density of the epoxy ring, making it more reactive.^28^

One of the other two peaks could be related to etherification. Etherification was induced in Flashed-FDE/PACM and 52 h.-FDE/PACM by using 10% less amine than the stoichiometric ratio of 1:2 amine: epoxy, and the data can be found in Figure S9. Flashed-FDE/PACM had a minor peak that could be related to etherification. However, the peak in Flashed-FDE/PACM did not correspond to the additional peaks in 52 h.-FDE/PACM, suggesting a lack of etherification. The additional reactions occurring require further characterization, but it is hypothesized that they are associated with cross-linking based on the increased *T*_g_. 52 h.-FDE/PACM had a *T*_g_ of 177 °C, while Flashed-FDE/PACM had a *T*_g_ of 79 °C.

Attenuated total reflection (ATR)-FTIR in the mid-IR range complemented the DSC experiments and was used to determine the cause of the additional exothermic peaks in the 52 h.-FDE/PACM system. An overview of the curing behavior is found in Figure S10. The ATR-FTIR data supported the presence of chlorine accelerating the epoxy-amine reaction and confirmed that furan rings participated in curing. However, the exact mechanism of how the furan rings participated was unclear. The furan rings started to open when the epoxy achieved a 60% conversion, indicating that amine was available in the system. Furthermore, after the furan rings started to open, the presence of ether, carbonyl, amide, and amine salt peaks was observed, and the hydroxyl and carbon-chlorine peaks started to decrease in intensity. However, the FTIR data do not provide sufficient information to fully understand the curing reactions of the 52 h.-FDE/PACM network due to overlapping peaks within the carbonyl region.

### Model Study of 52 h.-FDE Cured with Furfuryl Amine

^1^H- and ^13^C­{^1^H}-NMR techniques are better suited than FTIR for elucidating the bonds forming after furan ring opening. However, polymers that can be dissolved in solvents are required to use these techniques. Therefore, 52 h.-FDE was cured with furfuryl amine, a monoamine, to control network formation and allow the samples to be soluble in NMR solvent. Figure 8 shows reaction information for 52 h.-FDE cured with furfuryl amine, including A) a general reaction scheme, B) FTIR data, C) ^13^C­{^1^H}-NMR data, and D) ^1^H NMR data for unreacted and cured samples.

**Figure 8:**
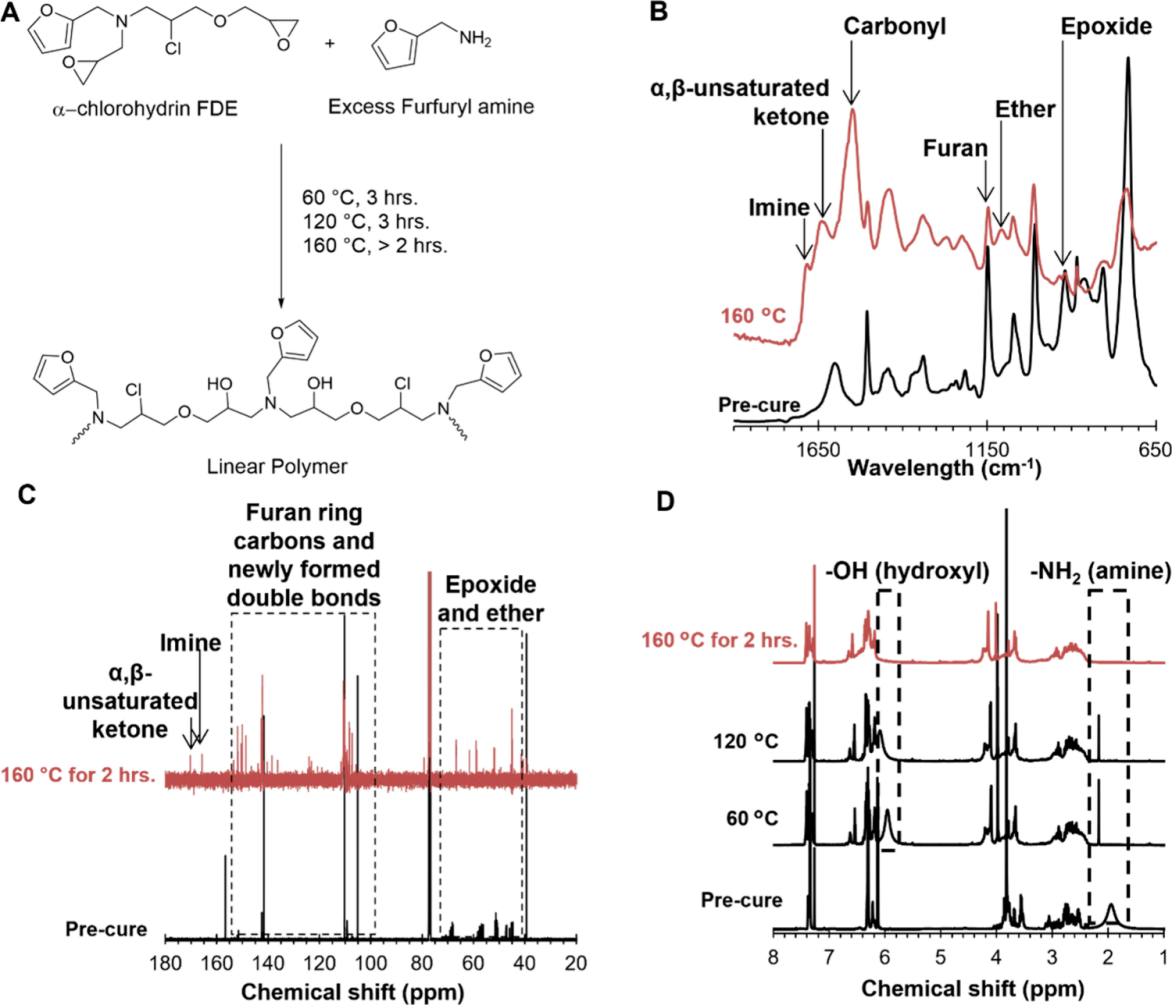
52 h.-FDE cured with furfuryl amine up to 160 °C. A) General reaction scheme. B) FTIR spectra before and after cure. C) ^13^C­{^1^H}-NMR at room temperature and 160 °C after 2 h. D) ^1^H NMR at room temperature and 160 °C after 2 h.

Based on the FTIR data, the epoxy peak (916 cm ^–1^) decreased as expected after being heated to 160 °C for 12 h. The furan ring peak (1012 cm ^–1^) also decreased as the temperature increased. The clear formation of a carbonyl peak (1555 cm^–1^) and α, ß-unsaturated ketone (1642 cm^–1^) confirmed the furan ring opening as the temperature increased. Additional peaks of ether (1110 cm^–1^) and imine (1685 cm^–1^) suggest that open furan rings react further to participate in the network formation.^29,30^ The formation of imine bonds suggests carbonyls react with available amine.

Similar results were observed in the ^13^C­{^1^H}-NMR data in Figure 8C, particularly for the formation of α, ß-unsaturated ketone and imine bonds. The samples for ^13^C­{^1^H}-NMR were taken after 2 h at 160 °C because beyond 2 h the samples were insoluble in the solvent for testing. Epoxy peaks (44–67 ppm) decreased as the reaction progressed, indicating an epoxy-amine reaction. The carbons associated with the furan ring (105–151 ppm) also decreased, and the appearance of α, ß-unsaturated ketones (170.2 ppm) supported the furan ring opening observed in the FTIR data. The imine peak (165.7 ppm) also showed that the open furan ring further reacts to participate in network formation through the reaction between newly formed carbonyls and primary amine.^29^

^1^H NMR, shown in Figure 8D, provided further insight into the furan ring behavior. The peak at 5.97–6.08 ppm was potentially a hydroxyl peak and formed at 60 °C from the epoxy-amine reaction.^29^ As the temperature increased to 160 °C, the hydroxyl peak unexpectedly disappeared, indicating a reaction occurred that involved this group. The amine peak (1.75–1.98 ppm) was also monitored, and at 120 °C, there was available amine, which supported the results from the FTIR.^29^ After a complete epoxy-amine reaction, the amine peak disappeared despite using an excess of furfuryl amine.

### Proposed Reaction Pathway for Participation of Furan Ring in Network Formation

The observations from DSC, FTIR, ^1^H-, and ^13^C­{^1^H}-NMR data were utilized to propose a potential mechanism for the furan ring behavior in 52 h.-FDE when cured with an amine. The chlorine on the epoxy monomer influenced the additional reactions observed in DSC, as Flashed-FDE did not exhibit similar polymerization reactions. The significant findings included furan ring opening on FTIR, formation of carbonyl, α, ß-unsaturated ketones, and imine bonds, as well as the potential removal of hydroxyl groups. These findings are crucial in understanding the complex behavior of the furan ring in 52 h.-FDE when cured with an amine. Based on these findings, a reaction pathway was proposed for curing 52 h.-FDE with an amine curing agent, which is demonstrated in Figure 9 using furfuryl amine as the curing agent.

**Figure 9:**
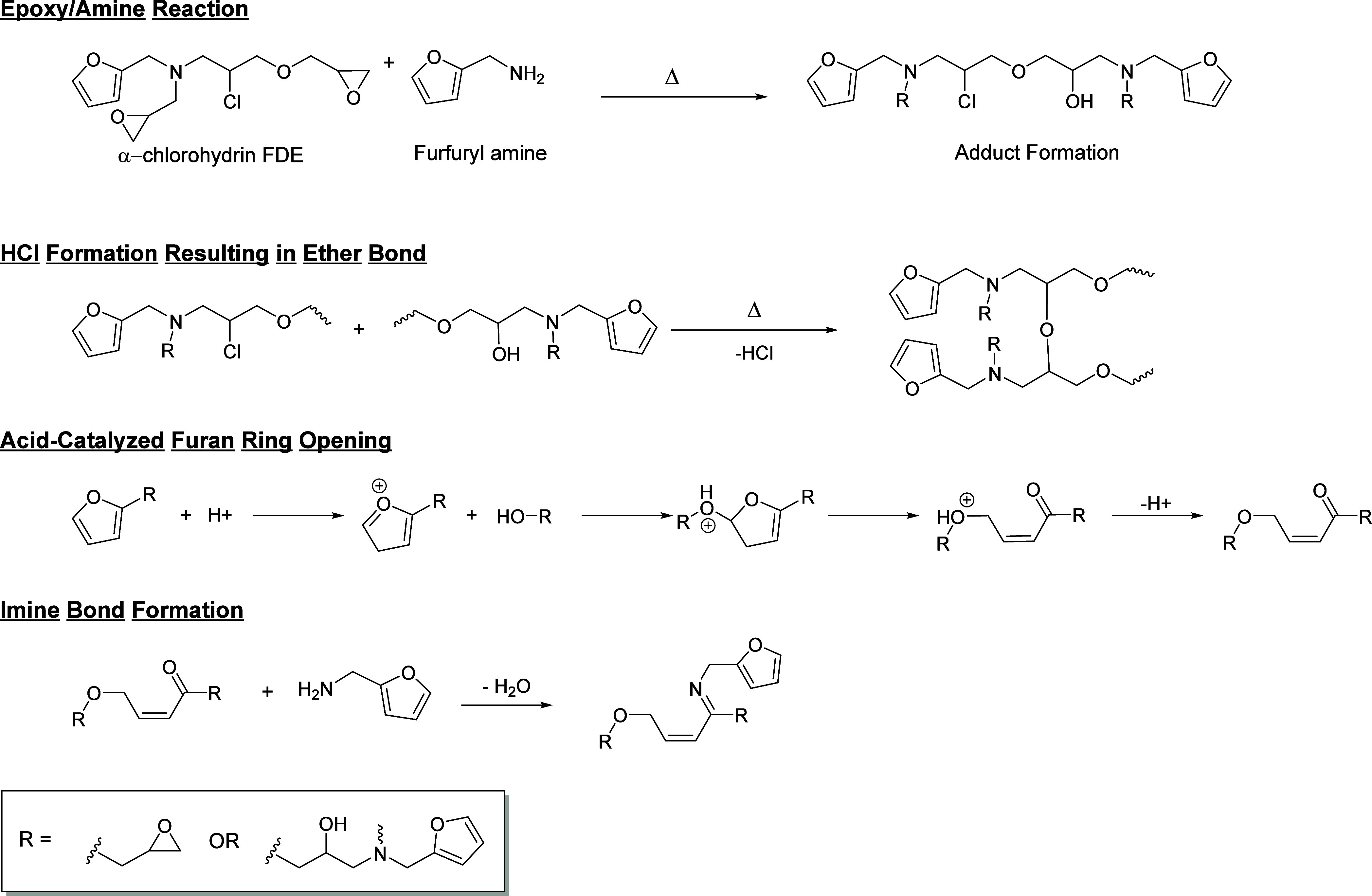
Proposed pathway for additional cross-linking reactions in 52 h.-FDE/amine through furan ring opening. Chlorine on the epoxy monomer enhances the reactivity of the hydroxyl to induce furan ring opening and create ether and imine bonds.

The initial step involves the reaction of 52 h.-FDE with the primary amine on furfuryl amine to produce a hydroxyl and a secondary amine, following typical epoxy-amine polymerization. It is hypothesized that the hydroxyl reacts with the chlorine on another molecule in an acid–base reaction to form an ether bond, which is a new cross-link and catalytic amounts of hydrochloric acid (HCl). The combination of HCl and elevated temperature could lead to acid-catalyzed furan ring opening.^31,32^ Commonly, acid-catalyzed furan ring opening occurs in the presence of water or methanol, but in this case, a hydroxyl on another polymer chain could act in the place of water or methanol. When the furan ring opens, an additional ether bond forms, acting as a second new cross-link, as well as an α, ß-unsaturated ketone and carbonyl. The newly formed carbonyl has the potential to react with the remaining amine in the system to form imine bonds, a third new cross-link.^30^ HCl is likely stabilized by the presence of the tertiary amines within the system to form ammonium salts.^33^

The molecular structure differences between Flashed-FDE and 52 h.-FDE played a significant role in the epoxy-amine polymerization. The chlorine on 52 h.-FDE promoted the formation of additional cross-links through the hydroxyl/chlorine reaction, acid-catalyzed furan ring opening reactions, and subsequent imine forming reactions. These reactions have led to the formation of carbonyls, α,ß-unsaturated ketones, ethers, and imine bonds, with the ether and imine bonds acting as cross-links. The absence of furan ring opening reactions in Flashed-FDE demonstrated the commonly accepted knowledge of the furan ring being a relatively stable functional group. The confirmation of these new cross-links will be established using cross-link density measurements in the subsequent section. The effect of the new cross-links on the physical properties of Flashed-FDE, 52 h.-FDE, and the FDE resins with varying reaction times cured with PACM will also be evaluated.

### Effect of Cross-Linking on Physical Properties

Using DMA, the storage modulus (E′) at 25 °C, the *T*_g_ as the peak of the loss modulus (E″), and the rubbery modulus at the rubbery temperature defined as the *T*_g_ plus 30 °C were measured for Flashed-FDE/PACM and 52 h.-FDE/PACM. Figure 10 includes the DMA thermograms for Flashed-FDE/PACM and 52 h.-FDE/PACM, where A) shows E′ as a function of temperature with an insert of the rubbery plateau and B) shows E″ as a function of temperature. Table 3 lists the DMA data for all samples cured with PACM. Figure S11 includes the tan δ versus temperature for Flashed-FDE/PACM and 52 h.-FDE/PACM. E′ represents the material’s elastic response and measures the amount of energy stored during deformation. E″ represents the material’s viscous behavior and measures the energy dissipated as heat during deformation.^4,34–36^

**Figure 10:**
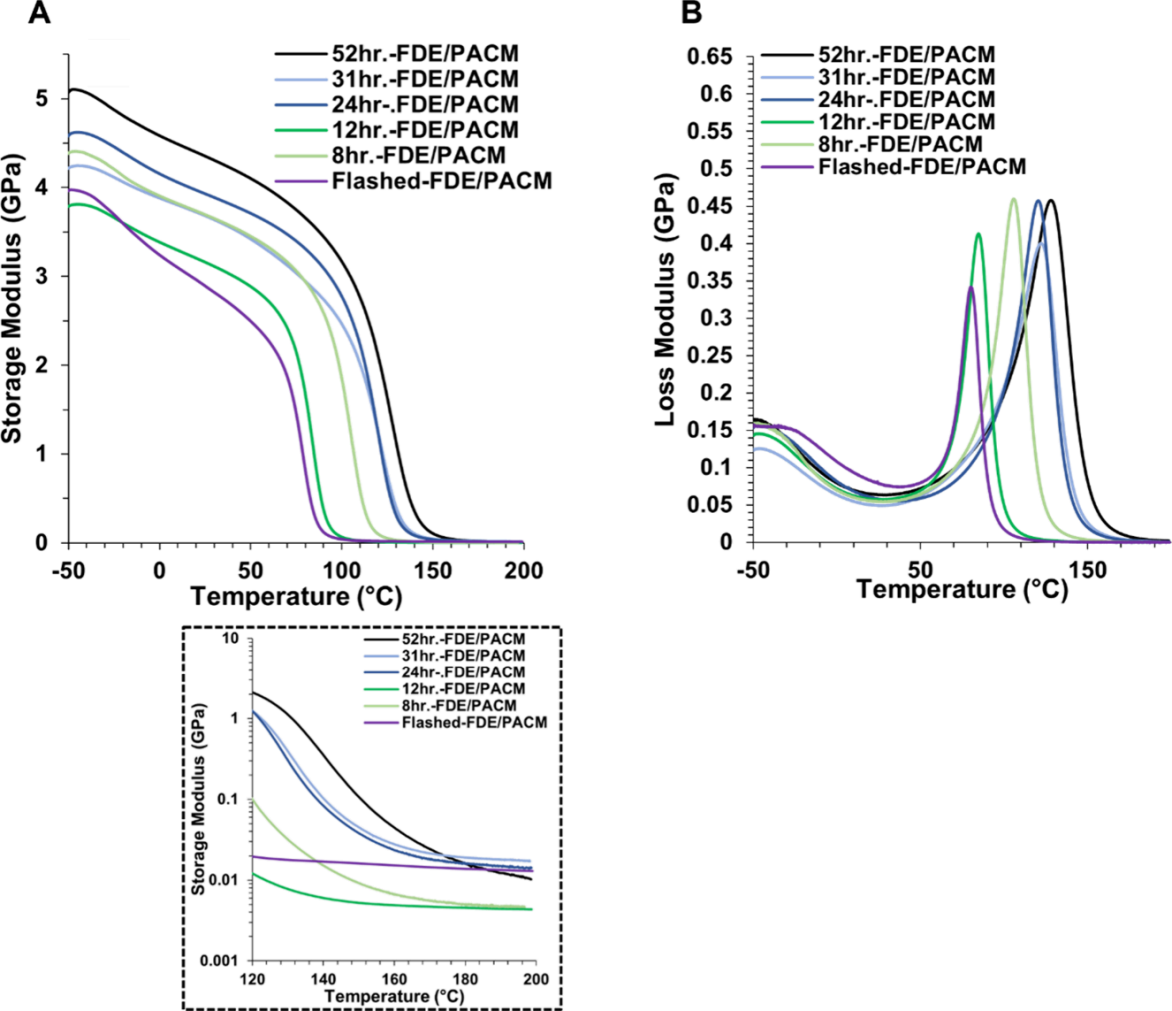
DMA thermograms for 1:10-FDE with increasing reaction time cured with PACM compared to Flashed-FDE/PACM. A) E′ as a function of temperature with an insert of the rubbery plateau region. B) E″ as a function of temperature.

**Table 3:** DMA Data for All FDE Systems Cured with PACM

system with PACM	*E*′ at 25 °C (GPa)	*T*_g_ (°C)	rubbery modulus (MPa)	ρ at 25 °C (g cm^–3^)	*v_e_* (mol cm^–3^)	theoretical *v_e_* (mol cm^–3^)
8 h.-FDE	3.69	106	19.9	1.251	1.95 × 10^–3^	
12 h.-FDE	3.11	85	16.4	1.211	1.69 × 10^–3^	
24 h.-FDE	3.93	120	39.9	1.221	3.78 × 10^–3^	
31 h.-FDE	3.68	122	39.9	1.219	3.76 × 10^–3^	
52 h.-FDE	4.35	129	47.8	1.251	4.43 × 10^–3^	3.05 × 10^–3^
Flashed-FDE	2.89	80	24.6	1.242	2.57 × 10^–3^	3.97 × 10^–3^

Flashed-FDE/PACM had an E′ at 25 °C of 2.89 GPa, while 52 h.-FDE/PACM had an increased E′ at 25 °C of 4.35 GPa. The increased E′ at 25 °C could be explained by the chain packing, which can be observed through the room temperature densities.^37^ The density of 52 h.-FDE/PACM was 1.251 g cm^–3^, while the density of Flashed-FDE/PACM was 1.242 g cm^–3^. Although the density of 52 h.-FDE/PACM is greater than that of Flashed-FDE/PACM, the improved chain packing may not be the sole cause of the increase. 52 h.-FDE/PACM contains a heavy chlorine atom that is likely contributing to the increased room temperature density.

*T*_g_ was measured as the peak of E″, which is considered a conservative value.^34,38^ *T*_g_ for Flashed-FDE/PACM was 80 °C, and for 52 h.-FDE/PACM, it was 129 °C. The increased *T*_g_ value for 52 h.-FDE/PACM revealed the difference in curing between a chlorinated and nonchlorinated epoxy containing furan rings. 52 h.-FDE/PACM created additional cross-links from the carbonyl-amine reaction and hydroxyl-furan reaction. Additional cross-links restrict chain mobility and increase the temperature when the material goes from a glassy state to a rubbery state.^39^ The amine stoichiometry also affected the ability to cross-link. All samples were cured assuming a 1:2 amine: epoxy ratio based on the theoretical value of Flashed-FDE. As more α-chlorohydrin FDE forms, as in 52 h.-FDE, less amine is required to fully react with epoxy, leading to excess amine being available during cure. The excess amine will participate in carbonyl-amine reactions, leading to an improvement in cross-linking density. Quantitatively, the cross-linking density (*v*_*e*_) confirmed the proposed additional cross-links forming for 52 h.-FDE/PACM. It should be noted that there was a difference in the *T*_g_ values compared to those measured by DSC, especially for 52 h.-FDE/PACM. The discrepancy was attributed to the use of different curing procedures. The DSC heated the sample at 1 °C min^–1^ to 250 °C, whereas the samples for the DMA were cured at 60 °C for 2 h, 120 °C for 2 h, and 160 °C for 3 h. The differences in curing affected the network formation, resulting in differing *T*_g_ values.

The theoretical *v*_*e*_ was calculated using the method described in Hill et al., assuming only epoxy cross-linking.^40^ For Flashed-FDE/PACM, *v*_*e*_ was calculated to be 3.97 × 10^–3^ mol cm^–3^, while for 52 h.-FDE/PACM, it was 3.05 × 10^–3^ mol cm^–3^. Based on the higher calculated *v*_*e*_, ideally, Flashed-FDE/PACM should create more cross-links. The *v*_*e*_ values were experimentally estimated using the Theory of Rubber Elasticity. The Theory of Rubber Elasticity was originally derived for branched and lightly cross-linked polymers, but literature has shown it is effective for highly cross-linked systems as well.^24^ Comparing the two systems, *v*_*e*_ for Flashed-FDE/PACM was 2.57 × 10^–3^ mol cm^–3^ while for 52 h.-FDE/PACM, it was 4.43 × 10^–3^ mol cm^–3^. 52 h.-FDE/PACM did not follow the expected trend and had a higher *v*_*e*_ than Flashed-FDE/PACM. It also had a higher experimental *v*_*e*_ than both theoretical values, suggesting the existence of additional cross-links. The increased *v*_*e*_ for 52 h.-FDE/PACM supported the new cross-links from the furan ring, which participated in network formation. Without chlorine in the system, additional cross-links could not form, as seen by the lower *v*_*e*_ in Flashed-FDE/PACM.

The viscoelastic properties were also evaluated for 1:10-FDE epoxidized products cured with PACM prepared from aliquots taken at different times during the adduct formation process. Each aliquot was found to contain different compositions of FDE, α-chlorohydrin FDE, and high molecular weight oligomers, as shown in the GPC data. The aliquot taken after 52 h resulted in mostly α-chlorohydrin FDE. Figure 10 also includes the DMA thermograms for the 1:10-FDE resins with increasing reaction time, where A) is E′ versus temperature with an insert of the rubbery plateau and B) is E″ versus temperature. Table 3 also lists the DMA data for all samples cured with PACM. Figure S11 also includes the tan δ versus temperature for all aliquots cured with PACM. There was no clear trend in E′ for the aliquots. However, there was a difference between the shortest reaction time of 8 h and the longest reaction time of 52 h. 8 h.-FDE/PACM had an E′ of 3.69 GPa, while 52 h.-FDE/PACM had an E′ of 4.35 GPa. The reaction times of 12, 24, and 31 h did not follow a trend. The lack of trend was explained by the GPC data. From 12 to 31 h, the concentrations of FDE, α-chlorohydrin FDE, and high molecular weight oligomers changed the most. The ratio of each component played a crucial role in determining the final properties, contributing to the inconsistencies.

There was a trend for *T*_g_ in the aliquot systems. Generally, as the reaction time increased, the *T*_g_ also increased, except for 12 h. 8 h.-FDE/PACM had the lowest *T*_g_ of 106 °C, and 52 h.-FDE/PACM had the highest of 129 °C. As more α-chlorohydrin FDE, or 52 h.-FDE, formed with increasing reaction time, more cross-links could form from the chlorine promoting acid-catalyzed furan ring opening. Additionally, as the reaction time increased, the amount of available amine to participate in the carbonyl-amine reaction increased. All samples were cured assuming a ratio of 1:2 amine: epoxy. 12 h.-FDE/PACM did not follow the trend and had the lowest *T*_g_ values of all the tested systems. The decreased properties were attributed to the increased high molecular weight oligomers found in the resin based on GPC data. 12 h.-FDE/PACM had similar values to Flashed-FDE/PACM but with an increased E′. Depending on the composition of each component, the properties can be tuned by changing the synthesis reaction time. Overall, the *T*_g_ increased with increasing reaction time and increasing α-chlorohydrin FDE content. As previously shown, α-chlorohydrin FDE created additional cross-links that led to increased viscoelastic properties, which were confirmed by calculating *v*_*e*_.

For *v*_*e*_, a similar trend was observed, where 8 h.-FDE/PACM had the lowest *v*_*e*_ of 1.95 × 10^–3^ mol cm^–3^ and 52 h.-FDE/PACM had the highest *v*_*e*_ of 4.43 × 10^–3^ mol cm^–3^. However, the trend was observed below 160 °C. Beyond 160 °C, 52 h.-FDE/PACM had a decreasing E′ in the rubbery region, which could result from the sample being extremely cross-linked, leading to crack formation during testing.

The additional cross-links formed in 52 h.-FDE/PACM led to increased properties compared to Flashed-FDE/PACM. Additionally, the epoxidized products obtained from aliquots taken at different times during adduct formation revealed that the properties could be altered depending on the resin composition by changing the synthesis reaction time. More α-chlorohydrin FDE improved viscoelastic properties because of the unique combination of chlorine with furan rings. 12 h.-FDE/PACM showed similar results to Flashed-FDE/PACM, except with a higher E′. Depending on the desired properties, the composition of the resin can be altered by changing the reaction time.

## Conclusions

In this study, the synthesis, polymerization, and physical properties of the epoxidation of furfuryl amine with epichlorohydrin to create FDE were investigated. First, the synthesis and isolation of FDE were studied by varying the reaction conditions, including epichlorohydrin concentration and epoxy adduct formation time. When furfuryl amine was epoxidized with excess epichlorohydrin, a chlorinated side product formed in addition to the desired FDE monomer. The chlorinated side product was α-chlorohydrin FDE, referred to as 52 h.-FDE in this work.

The next step was to evaluate the effect of structural variations on epoxy-amine polymerization by comparing the polymerization of Flashed-FDE and 52 h.-FDE cured with PACM. Curing 52 h.-FDE with PACM promoted the formation of additional cross-linking reactions. A reaction pathway was proposed for the formation of additional cross-links. The hydrolyzable chlorine on 52 h.-FDE accelerated the epoxy-amine polymerization but also promoted the ring opening of furan. Furan is commonly accepted as a nonreactive group, but under specific conditions, it will act as a functional group to participate in network formation.

Finally, the increased cross-links were confirmed using cross-link density measurements, and the effect of additional cross-links on physical properties was evaluated. The experimental *v*_*e*_ for 52 h.-FDE/PACM was greater than the experimental *v*_*e*_ for Flashed-FDE/PACM, as well as the theoretical *v*_*e*_ values. The increase *v*_*e*_ supported the findings from the second step. The additional cross-linking resulted in an E′ of 4.4 GPa and *T*_g_ of 129 °C for 52 h.-FDE/PACM. Epoxy resins containing both a furan ring and hydrolyzable chlorine have the potential to increase the final properties due to the additional cross-linking reactions the furan ring can participate in.

## Supplementary Material



## Notes

The Supporting Information includes additional experimental details and methods, such as the synthesis of FGE, detailed GPC data, full spectra for near-IR, ^1^H NMR, and ^13^C­{^1^H}-NMR, further development of the curing mechanism using DSC, calculated mass loss during cure, heated stage FTIR study, and the tan δ as a function of temperature for all cured samples (PDF)
