# Supplementary material for: Synthesis, Processing, and Performance of a Furan-Based Glycidyl Amine Epoxy Resin
Source: ACS Omega. 2025 Nov 13;10(46):55432–45. doi: 10.1021/acsomega.5c05822 (PMC12658604; doi:10.1021/acsomega.5c05822)
Supplement: Supplementary file 1 [file ao5c05822_si_001.pdf]

SUPPORTING INFORMATION

Synthesis, processing, and performance of a furan-based, glycidyl amine epoxy resin

*Amy Honnig Bassett, Emre Kinaci, Giuseppe R. Palmese**

Rowan University, Department of Chemical Engineering, Advanced Materials and Manufacturing Institute (AMMI), 201 Mullica Hill Rd., Glassboro, NJ 08028, USA

*Corresponding author, palmese@rowan.edu

Table of Contents

Experimental....1
Results....4
GPC Data for 1:10 FDE Aliquots....4
Near-IR Spectra for the Reaction Kinetics of 1:10 FDE....5
^{1}H-NMR Analysis for 1:10 FDE Aliquots Compared to Flashed-FDE....6
^{13}C-NMR Full Spectra for 1:10 FDE Aliquots Compared to Flashed FDE....10
Synthesis of Furfuryl Glycidyl Ether (FGE)....12
DSC Analysis for Curing Mechanism Development....13
Monitoring the Cure of Flashed-FDE and 52hr.-FDE with PACM....16
DMA Data for All FDE Samples....19
References....20

Experimental

Synthesis for Furfuryl Glycidyl Ether (FGE)

Furfuryl alcohol was epoxidized using epichlorohydrin. A molar ratio of 1:5 furfuryl alcohol: epichlorohydrin was used, which matched the molar ratio used in the 1:10 FDE reaction. Furfuryl alcohol (20.0 g), epichlorohydrin (94.3 g), and tetraethylammonium bromide (1.14 g) were added to a 1000 mL 4 necked- round bottom flask. The flask had a constant pressure dropping funnel, overhead mixer, thermometer, and condenser. The mixture was reacted for 52 h

at room temperature to confirm the unique nature of glycidation of an amine. After 52 h, the reaction mixture was cooled to 0 °C in an ice bath before adding 50 % w/w sodium hydroxide. The reaction mixture was allowed to react for 2 h after dropping in sodium hydroxide at a slight heat of 60 °C. The mixture was then dissolved in ethyl acetate and washed twice with brine. A rotary evaporator was used to remove ethyl acetate and unreacted epichlorohydrin. The final product was amber in color and of low viscosity. FGE was purchased from Acros Organics to compare the laboratory made FGE.

Epoxy Equivalent Weight (EEW) Measurement

Epoxies were methacrylated using excess methacrylic acid. FDE (20.0 g), an estimated 20 % excess methacrylic acid (19.86 g), and AMC-2 catalyst (0.2 g) were added to a 250 mL 1-necked round bottom flask with a magnetic stirrer and reacted at 80 °C for 4 h. After 4 h, a 1 g sample was titrated as described in La Scala et al. and is referred to as the titrated sample.[1] The remaining percentage and mass of free acid in the titrated sample were calculated using Equations 1 and 2. From the mass of the remaining free acid in the titrated sample, the mass of the free methacrylic acid in the overall methacrylation reaction was calculated using Equation 3. This value was then used to calculate the mass of methacrylic acid consumed in the overall reaction using Equation 4. Finally, the EEW was calculated by knowing the mass of methacrylic acid consumed in the overall reaction, the mass of starting epoxy in the overall methacrylation reaction, and the molecular weight of methacrylic acid, as shown in Equation 5. Below is an example calculation of how the EEW was calculated for Flashed-FDE.

Table S1: Known values for the newly developed EEW methodology

Purpose	Name	Variable	Value	Unit
Acid Number Titration Values	Mass of titrated sample	m_S	1.0277	g
	Volume of NaOH	V	2.9	mL
Overall Methacrylation Reaction Values	Initial mass of epoxy	m_E	20.0	g
	Initial mass of methacrylic acid	m_M	19.86	g
Constants	Molarity of NaOH solution	N	0.5	M
	Molecular weight of NaOH	MW_{NaOH}	40.00	g/mol
	Molecular weight of methacrylic acid	MW_{MA}	86.06	g/mol

Calculate the acid number using Equation 1.

$$Acid\ Number = \frac{N(MW_{MA})\,(V)}{m_s} = \frac{0.5\ M\left(86.06\,\frac{g}{mol}\right)(2.9\ mL)}{1.0277\ g} = 56.4\,\frac{mg_{NaOH}}{g_{sample}}$$

From the acid number, the mass of acid remaining in the titrated sample (m_{at}) can be calculated using Equation 2.

$$m_{at} = \frac{acid\ number}{40{,}000\,\frac{mg_{NaOH}}{mol}} = \frac{56.4\,\frac{mg_{NaOH}}{g_{sample}}}{40{,}000\,\frac{mg_{NaOH}}{mol}} =$$

Knowing the mass of acid remaining in the titrated sample (m_{at}), the mass of acid remaining in the overall methacrylation reaction (m_R) can be calculated using Equation 3.

$$m_R = \left(\frac{m_E + m_M}{m_S}\right) m_{at} = \left(\frac{20.0\ g + 19.86\ g}{1.0277\ g}\right) 0.1213\ g =$$

The mass of acid consumed in the overall methacrylation reaction (m_C) can be calculated by knowing the starting methacrylic acid mass (m_M) and remaining acid mass in the overall reaction (m_R) using Equation 4.

$$m_C = m_M - m_R = 19.86\ g - 4.705\ g =$$

The EEW can be calculated by knowing the mass of acid consumed in the overall methacrylation reaction (m_C) using Equation 5.

$$EEW = \frac{m_E(MW_{MA})}{m_C} = \frac{(20.0\ g)\left(86.06\,\frac{g}{mol}\right)}{15.155\ g} = 113.6\,\frac{g}{eq}$$

Results

GPC Data for 1:10 FDE Aliquots

The GPC data, including the elution time and calculated concentration based on the peak areas for the aliquots of 1:10 FDE, are given in Table S2 and correspond to Figure 3.

Table S2: GPC data for increasing reaction time for 1:10 FDE

Reaction Time (h)	Elution Time (min)	Calculated Concentration (%)
2	7.987	15.2
	8.056	11.5
	8.250	7.4
	8.383	17.6
	8.480	36.8
	8.809	11.5
4	7.933	15.3
	8.104	28.4
	8.339	23.8
	8.490	25.6
	8.700	6.9
8	8.252	27.9
	8.417	64.1
	8.550	8.0
12	8.217	39.9
	8.444	39.5
	8.558	20.6
24	8.217	3.4
	8.444	78.4
	8.558	18.2
31	8.217	6.17
	8.443	93.8
52	8.217	5.4
	8.436	94.6

Near-IR Spectra for the Reaction Kinetics of 1:10 FDE

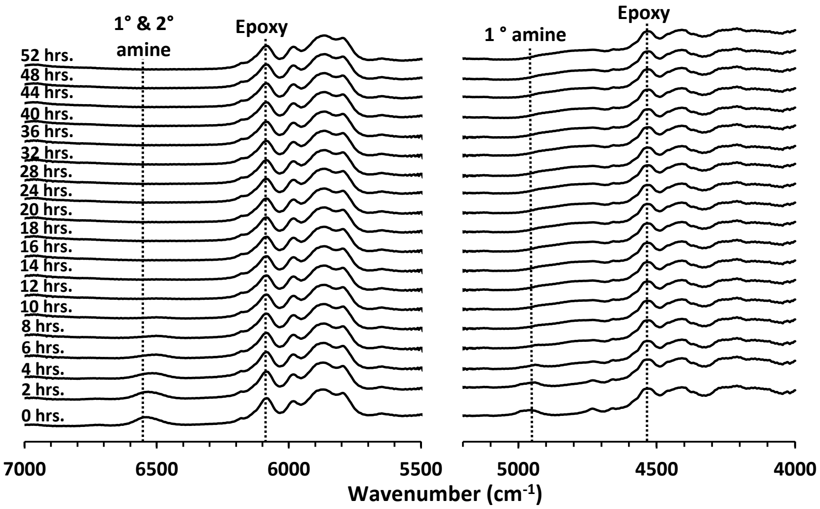

Figure S1: Near-IR spectra for reaction kinetics of 1:10 Furfuryl amine: epichlorohydrin with (1° and 2° amines (6570 cm^{-1}), epoxy overtones (6080 cm^{-1}), 1° amine (4940 cm^{-1}), and epoxy (4540 cm^{-1}) labeled.[2, 3] (Spectra offset for clarity)

^{1}H-NMR Analysis for 1:10 FDE Aliquots Compared to Flashed-FDE

All purified FDE samples were characterized by ^{1}H-NMR (24 °C in $CDCl_3$) using a Bruker Avance Neo 400 MHz NMR Spectrometer. Figure S2 is the ^{1}H-NMR data for purified FDE resins with A) as the full spectra, B) is from 6-7.5 ppm and related to the furan ring hydrogens, C) is from 3.3-4.5 ppm and related to the hydrogens connecting the furan ring to the nitrogen, and D) is from 2.2-3.4 ppm and related to the hydrogens on the epoxy rings.

The first and second regions (**Figure S2**B and C) showed that the products at 2 and 4 h did not resemble FDE based on peak broadening and the presence of additional peaks. Increasing the time up to 52 h showed the peaks to match Flashed-FDE. Therefore, the 52 h product contained some differences from Flashed-FDE but did have similarities, such as in the monosubstituted furan ring. The peaks from 3.3-3.7 ppm are associated with chlorinated products. As the reaction time increased, the peaks became more intense, suggesting a greater increase in chlorinated species. In Flashed-FDE, there are no peaks in this region, indicating a pure product.

The third region in **Figure S2**D is from 2.2-3.4 ppm and is related to the protons on the epoxy rings. In Flashed-FDE, there were six groups of split peaks, each associated with the protons on the two epoxy rings. The epoxy region further confirmed that the products at 2 and 4 h did not resemble FDE. The peaks in this region were broad and not well resolved in the splitting pattern seen in Flashed-FDE. The quartet from 2.3-2.4 ppm is one proton of the epoxies in FDE. Following the quartet was the easiest to track for FDE formation. Some FDE started forming at 8 h, as seen in the GPC trace. Increasing the time to 31 h showed FDE was still in the purified product. However, at 52 h, there was a decrease in the intensity of the quartet, which showed a drop in FDE formation. This same trend was seen in the GPC trace. At 52 h, there was one peak that did not line up with the FDE peak. Based on the other peaks in the epoxy hydrogen region, the conclusion can be drawn that the 52 h product is not FDE but some other epoxidized product. The epoxidized product could contain chlorine or a glycidyl ether. The additional peaks and broadness would arise from a different type of epoxy formation, such as a glycidyl ether.

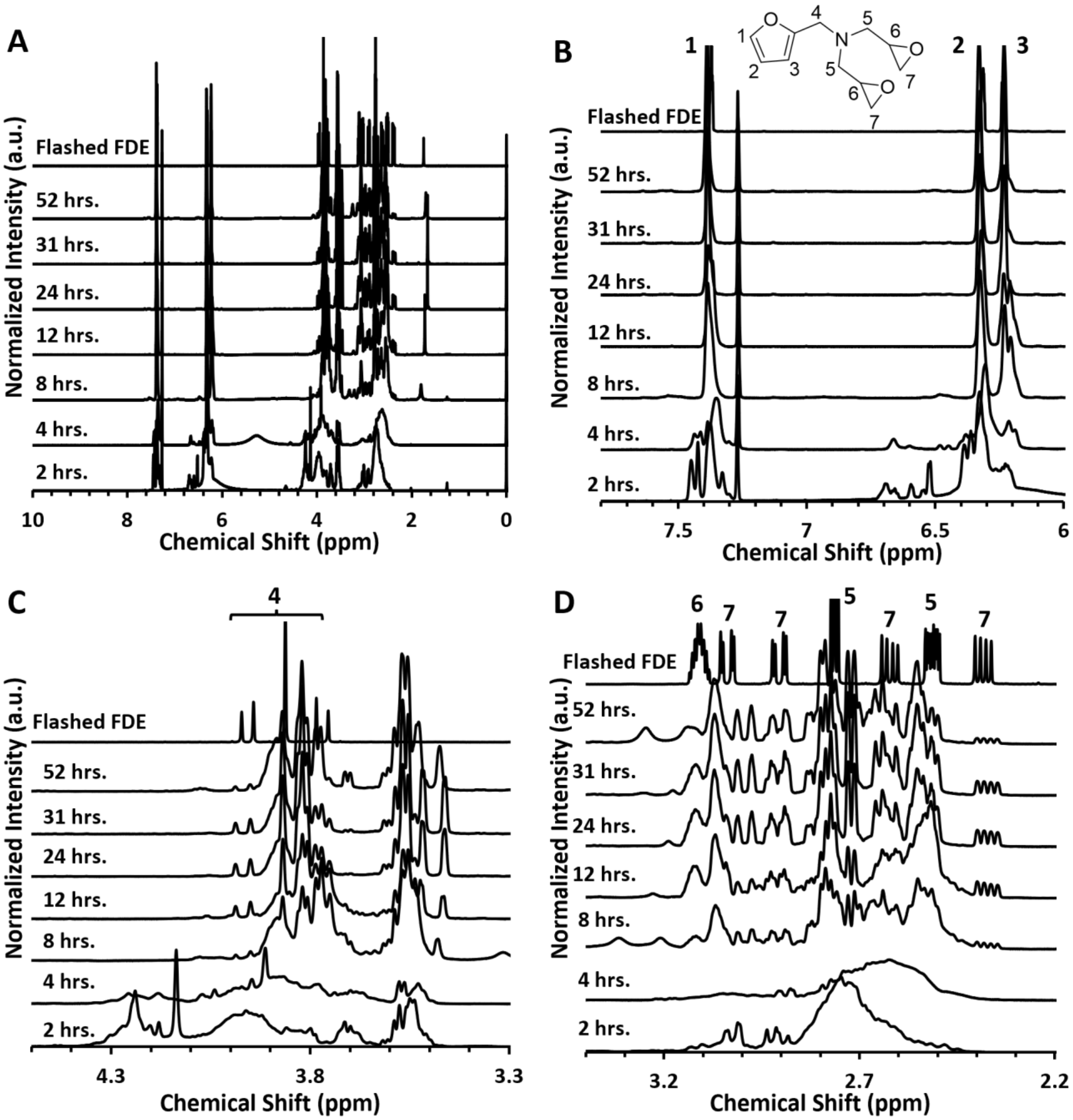

Figure S2: ^{1}H-NMR for increasing reaction time of 1:10 FDE compared to Flashed-FDE. A) full spectra, B) Furan ring region with FDE structure of labeled hydrogens in all three regions, C) connection between furan ring and epoxides, D) epoxide region

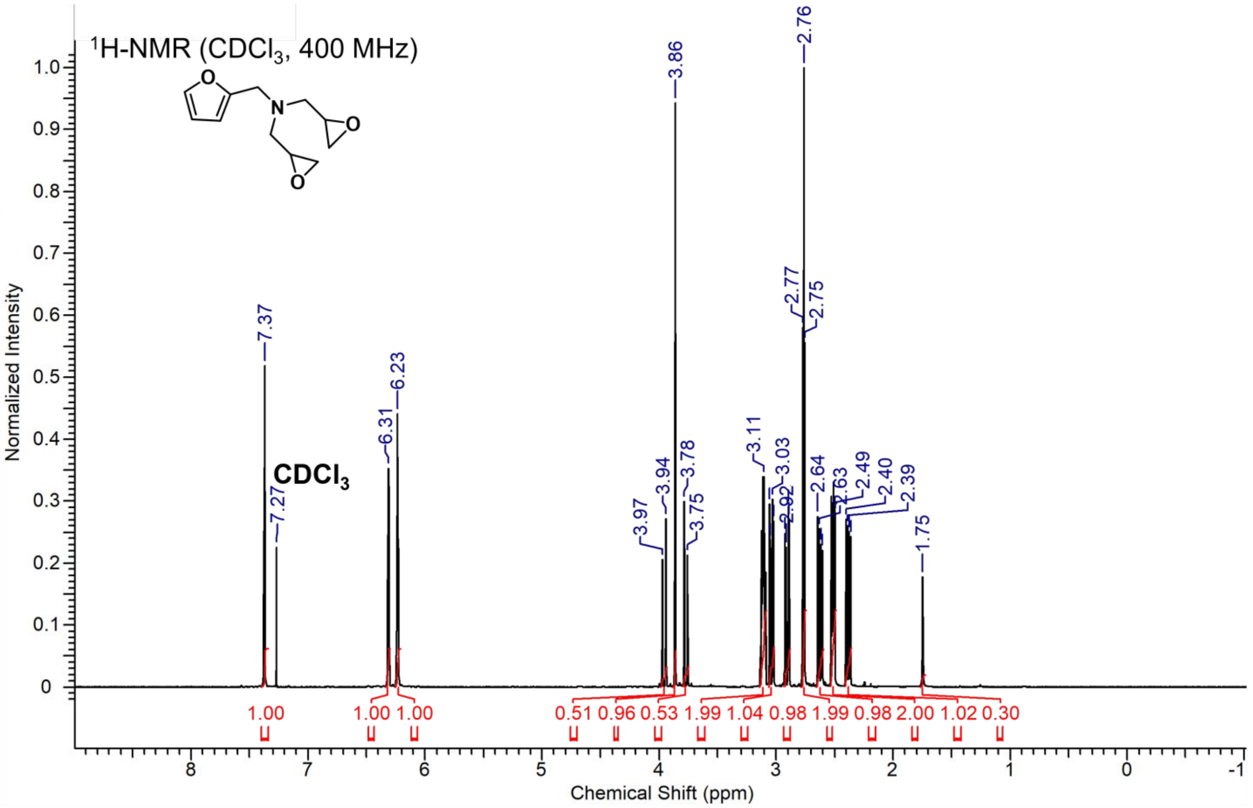

Figure S3: ^{1}H-NMR for Flashed-FDE with peak positions and integration

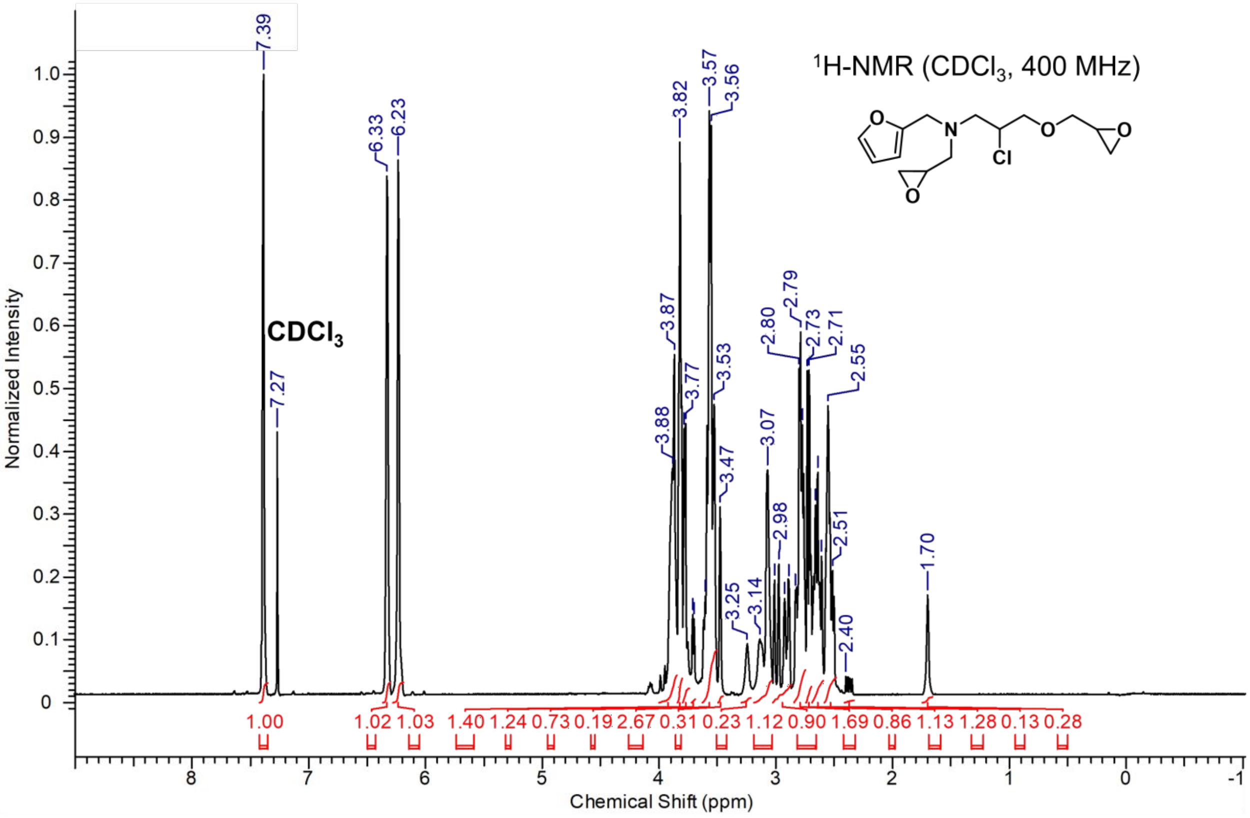

Figure S4: ^{1}H-NMR for 52hr.-FDE (α-chlorohydrin FDE) with peak positions and integration

$^{13}C\{^1H\}$-NMR Full Spectra for 1:10 FDE Aliquots Compared to Flashed FDE

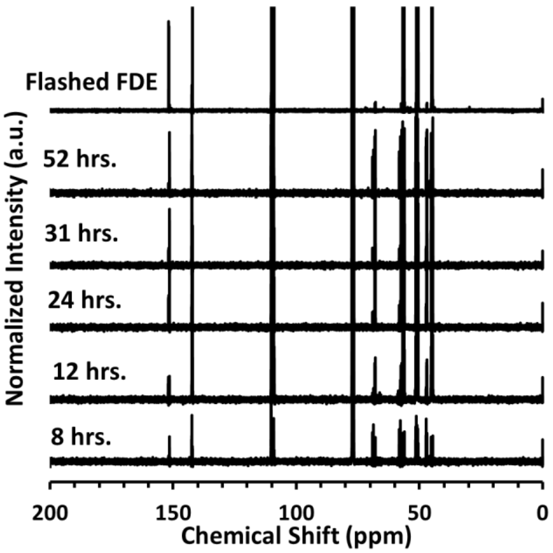

Figure S5: $^{13}C\{^1H\}$-NMR spectra for increasing reaction time of 1:10-FDE compared to Flashed-FDE

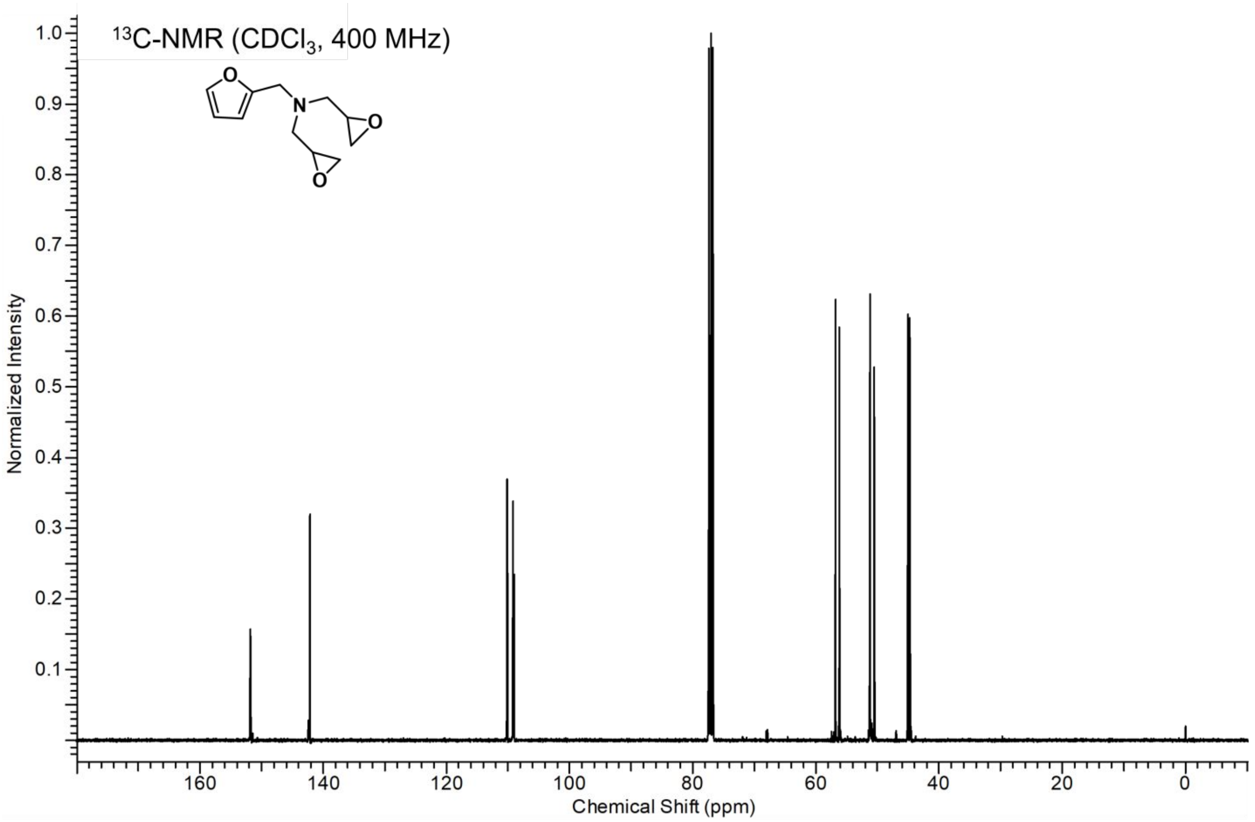

Figure S6: $^{13}C\{^1H\}$-NMR for Flashed-FDE

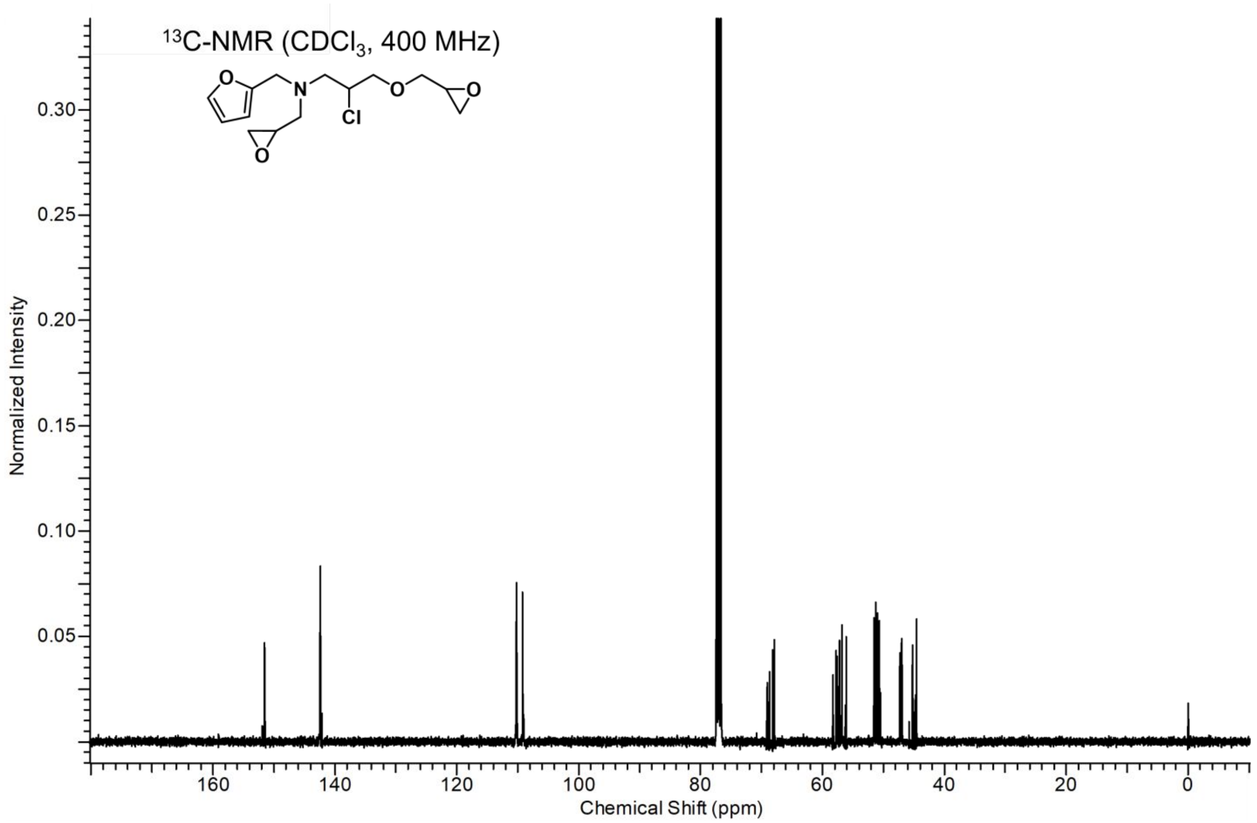

Figure S7: $^{13}C\{^{1}H\}$-NMR for 52hr.-FDE (α-chlorohydrin FDE)

Synthesis of Furfuryl Glycidyl Ether (FGE)

Figure S8 shows the GPC trace for FGE compared to Flashed-FDE, 12hr.-FDE to show the presence of α-chlorohydrin FDE, and a purchased FGE. For FGE, there was one major peak and one minor peak. The major peak was attributed to FGE, and the minor peak was associated with oligomers that inevitably form during epoxidation with epichlorohydrin. Despite the minor oligomer formation, FGE formed a single major peak at extended reaction times and did not initiate additional reactions, unlike those observed in the FDE synthesis.

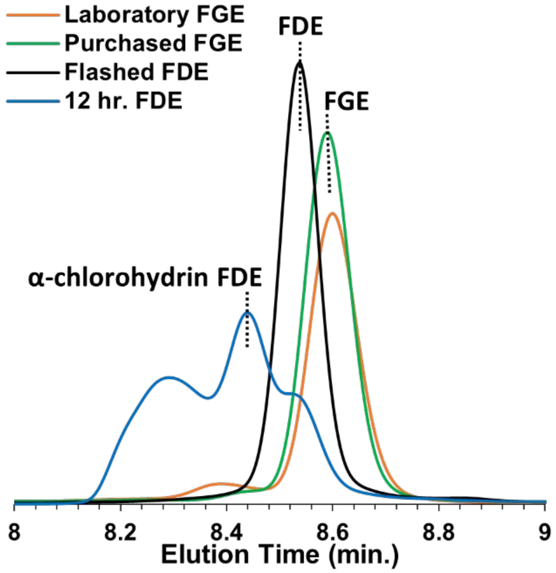

Figure S8: GPC trace for furfuryl glycidyl ether (FGE) compared to Flashed-FDE and 12hr.-FDE

DSC Analysis for Curing Mechanism Development

A TA Instruments DSC 2500 was used to observe the curing peaks when 10 % less amine was used. Flashed-FDE and 52hr.-FDE were cured with PACM at exact stoichiometry and 10 % below stoichiometry. The epoxy and amine were mixed using a Thinky ARE-310 planetary mixer for 1.5 min and defoamed for 1 min. Approximately 10 mg of the sample was placed into a Tzero pan with a Tzero Hermetic lid. The samples were heated from 0-250 °C at 1 °C min^{-1} in a nitrogen atmosphere and the thermograms are shown in Figure S9. The curing temperature was determined as the peak of the exotherm. Table S3 shows the peak enthalpies when Flashed-FDE/PACM and 52hr.-FDE/PACM are cured with a stoichiometric ratio of 1:2 amine: epoxy.

Table ***S4*** shows the peak enthalpies for Flashed-FDE/PACM and 52hr.-FDE/PACM when cured with 10 % less amine than the stochiometric ratio. When 10 % less amine was used in Flashed-FDE/PACM, a broad, exothermic peak was present with a peak temperature of about 100 °C, representing the epoxy-amine reaction. An additional peak with a peak temperature of about 200 °C was also observed. The additional peak could be epoxy homopolymerization because of the reduced amine amount.

52hr.-FDE/PACM, regardless of amine amount, displayed three exothermic peaks shown in **Figure S9**B. The third peak was likely not epoxy homopolymerization because it did not align with epoxy homopolymerization in Flashed-FDE/PACM, but rather another more exothermic reaction, such as furan ring degradation.

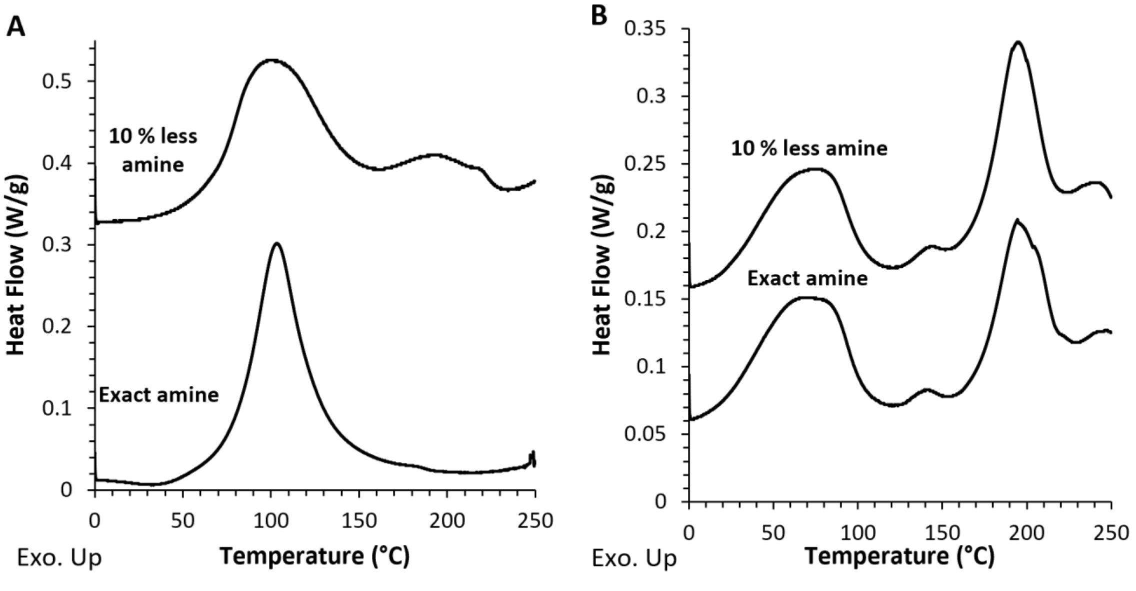

Figure S9: DSC thermograms for understanding the effect of amine stoichiometry on cure. A) Flashed-FDE/PACM. B) 52hr.-FDE/PACM

Table S3: DSC exothermic peak enthalpies cured using a stoichiometric ratio of 1:2 amine: epoxy

System	Peak 1 Enthalpy (J/g)	Peak 2 Enthalpy (J/g)	Peak 3 Enthalpy (J/g)
Flashed-FDE/PACM	647.31	-	-
52hr.-FDE/PACM	294.32	5.4648	189.21

Table S4: DSC exothermic peak enthalpies cured using 10 % less amine than the stoichiometric ratio of 1:2 amine: epoxy

System	Peak 1 Enthalpy (J/g)	Peak 2 Enthalpy (J/g)	Peak 3 Enthalpy (J/g)
Flashed-FDE/PACM	248.09	36.839	-
52hr.-FDE/PACM	268.22	4.2120	208.48

Mass Loss During Cure

Due to the furan ring opening, some mass loss was expected during the cure. The mass loss was calculated for Flashed-FDE and 52hr.-FDE cured with stoichiometric amounts of PACM. The curing procedure was 60 °C for 2 h, 120 °C for 2 h, and 160 °C for 2 h. The resin mass was measured before cure, and the polymer mass was measured after cure. The difference in the masses was calculated to be the mass loss during cure. Table S5 shows the percentage of mass loss during cure. 52hr.-FDE/PACM lost about 5 %, while Flashed-FDE/PACM lost 2 %. The difference in the mass losses was described by the furan ring opening. It is well known that volatiles are created when the furan ring opens.[4] The Near-IR data showed more furan ring conversion for 52hr.-FDE/PACM than for Flashed-FDE/PACM, as chlorine had an accelerating effect on the hydroxyl-furan reaction.

Table S5: Mass loss during cure of 52hr.-FDE and Flashed-FDE with PACM at the measured EEW values using the acid number titration method

System	Mass Loss During Cure (%)
52hr.-FDE/PACM	5
Flashed-FDE/PACM	2

Monitoring the Cure of Flashed-FDE and 52hr.-FDE with PACM

A heated stage FTIR study of Flashed-FDE and 52hr.-FDE with PACM was conducted to mimic the DSC ramp rate experiment and determine the bonds breaking and forming during polymerization. A Specac Golden Gate High Temperature ATR accessory was used with a Thermo Scientific Nicolet iS50 FTIR Spectrometer in the mid-IR range (650-4000 cm^{-1}). Absorbance mode was used to collect 32 scans with a 4 cm^{-1} resolution. Spectra were taken every 15 min while heating from 25-250 °C at 1 °C min^{-1}. Flashed-FDE and 52hr.-FDE were cured with PACM at the measured EEW values. The peak height of 1450 cm^{-1} was used as a reference. Conversion of epoxy (EP, 916 cm^{-1}) and furan (F, 1012 cm^{-1}) was calculated using Equation 6, where I(t) is the peak intensity during cure and I(t = 0) is the initial peak intensity:[5]

$$Conversion_{EP/F}\ (\%) = 1 - \left[\frac{I\ (t)_{\frac{EP/F}{ref}}}{I\ (t=0)_{\frac{EP}{F}}} \right]$$

Figure S10 presents an overview of the curing mechanism where A) is the conversion plot of epoxy and furan for Flashed-FDE/PACM with respect to cure temperature, B) is the heated stage FTIR spectra (off set for clarity) for Flashed-FDE/PACM, C) is the conversion plot of epoxy and furan for 52hr.-FDE/PACM with respect to cure temperature, and D) is the heated stage FTIR spectra (off set for clarity) for 52hr.-FDE/PACM. The calculated conversions are representative of the curing because the temperature is known to influence the peak intensities. The primary goal of using the heated FTIR stage was to gain insight into the bonds that were breaking and forming during the cure. The epoxy (916 cm^{-1}), furan ring (1011 cm^{-1}), carbonyl region (1600-1800 cm^{-1}), and hydroxyl (~3500 cm^{-1}) peaks were monitored.[6, 7] Additional peaks for carbon-chlorine (695 cm^{-1}) and amine salt (1550 cm^{-1}) were observed for 52hr.-FDE/PACM.[6, 8-10]

The conversion plots showed the accelerating effect of chlorine on the epoxy-amine reaction. **Figure S10**A is the conversion plot for Flashed-FDE/PACM, which shows that the epoxies started reacting between 35-45 °C, similar to the DSC. By 250 °C, the epoxy had reacted up to 80 %. The lower-than-desired epoxy conversion was attributed to the non-isothermal heating method used for both DSC and FTIR. **Figure S10**C is the conversion plot for 52 hr.-FDE/PACM and shows the epoxies started reacting between 15-25 °C, supporting the accelerating effect chlorine had on the epoxy-amine reaction. A halogen, such as chlorine, on the epoxy monomer accelerates the epoxy-amine reaction because a stronger polar bond is formed. Carbon-chlorine bonds are more polar than carbon-carbon bonds.[11]

The furan ring conversion was also tracked for both systems to understand its role in the network formation. Furan rings were not expected to participate in the network formation. For Flashed-FDE/PACM, the furan ring reacted up to 10 % at 240 °C (**Figure S10**A). The slight conversion of furan rings resulted from the high temperatures because furan rings are known to open at temperatures near 250 °C.[4, 7, 12] In Flashed-FDE/PACM, the furan ring did not participate in the network formation. For 52hr.-FDE/PACM, furan started to convert at 130 °C but rapidly reacted above 175 °C (**Figure S10**C). The final conversion was 54 % by 250 °C. Unlike Flashed-

FDE/PACM, 52hr.-FDE/PACM did not convert as a result of extreme temperature. Rather, the low-temperature furan ring conversion was attributed to participation in network formation.

The FTIR spectra give an idea of how the furan ring is reacting in 52hr.-FDE/PACM. **Figure S10**B is the FTIR spectra for Flashed-FDE/PACM. The expected behavior of the epoxy peak decreasing, the hydroxyl peak forming, and the furan peak remaining constant was observed. For 52hr.-FDE/PACM, the spectra, as shown in **Figure S10**D has the formation of critical peaks. The important aspects are the formation of imide peaks, carbonyls, amine salt, and the disappearance of hydroxyl, furan, epoxide, and carbon-chlorine. With the formation of carbonyl peaks and the disappearance of furan peaks, it was believed that the furan rings were opening to create carbonyl peaks. Furthermore, the formation of amine salts indicates a potential reaction to create hydrochloric acid (HCl) that is being stabilized to create amine salts. This corresponds well to the disappearance of the carbon-chlorine peak. The formation of an amide peak suggests that newly formed carbonyls are reacting with available amine to create an additional crosslink.

Based on the FTIR data, the chlorine is impacting the network formation and the behavior of the furan ring. The chlorine potentially reacts in an acid-base reaction to create HCl, as observed by the decrease in carbon-chlorine peak intensity and the formation of an amine salt peak. HCl has the potential to go through acid-catalyzed furan ring opening to create carbonyls. Furthermore, the newly formed carbonyls have the potential to react with available amine to create imine bonds.

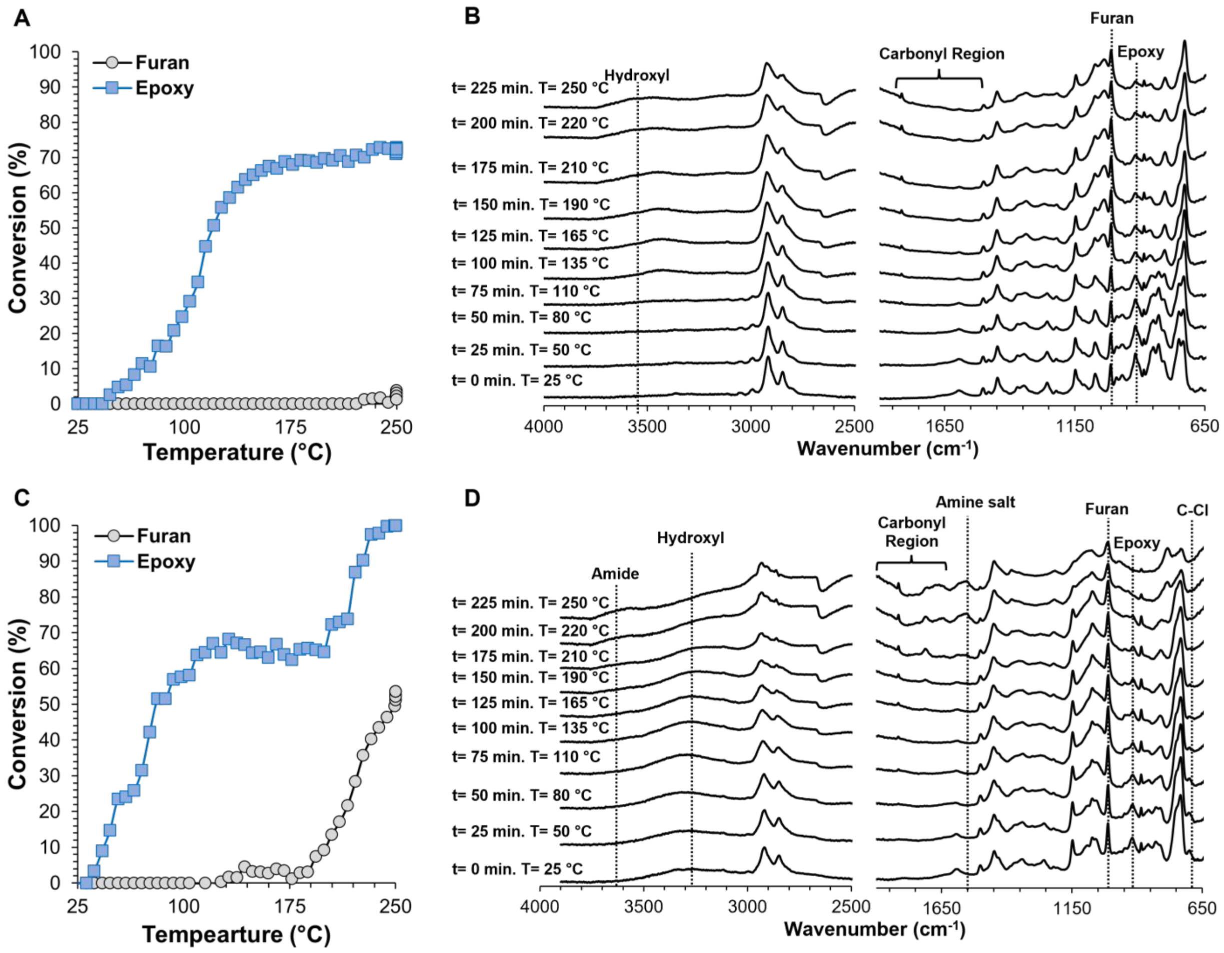

Figure S10: Curing mechanism development. A) Conversion versus temperature plot for Flashed- FDE/PACM. B) Heated stage FTIR spectra for Flashed-FDE/PACM (offset for clarity). C) Conversion versus temperature plot for 52hr.-FDE/PACM. D) Heated stage FTIR spectra for 52hr.-FDE/PACM (offset for clarity)

DMA Data for All FDE Samples

Tan δ provides an upper limit for the T_g and is shown in Figure S11. The same trend was observed for T_g based on tan δ as was seen for the loss modulus (E″). 52hr.-FDE/PACM had a higher T_g of 147 °C compared to 88 °C for Flashed-FDE/PACM. The increased T_g resulted from the additional crosslinks from the acid-catalyzed furan ring opening.

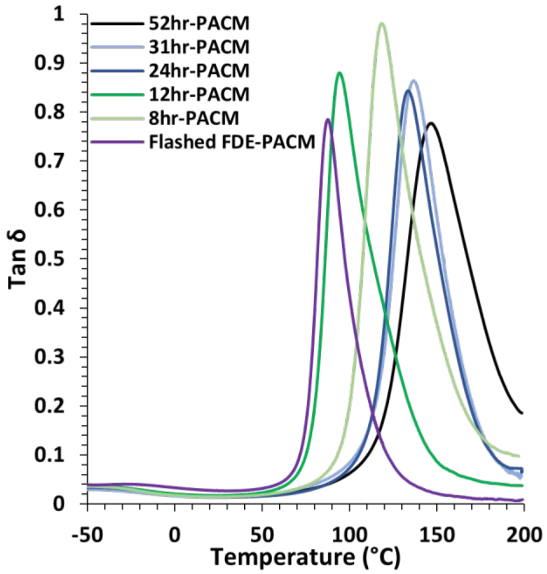

Figure S11: Tan δ for 1:10 FDE aliquots cured with PACM and compared to Flashed-FDE/PACM

References

(1) LaScala, J. J.; Sands, J. M.; Orlicki, J. A.; Robinette, E. J.; Palmese, G. R. Fatty Acid-Based Monomers as Styrene Replacements for Liquid Molding Resins. *Polymer* **2004**, *45*, 7729-7737.
(2) Mijovic, J.; Andelic, S. A Study of Reaction Kinetics by Near-Infrared Spectroscopy. 1. Comprehensive Analysis of a Model Epoxy/Amine System. *Macromolecules* **1995**, *28*, 2787-2796.
(3) Kinaci, E.; Can, E.; LaScala, J. J.; Palmese, G. R. Influence of Epoxidized Cardanol Functionality and Reactivity on Network Formation and Properties. *Polymers* **2020**, *12* (9), 1956.
(4) Fitzer, E.; Schäfer, W. The Effect of Crosslinking on the Formation of Glasslike Carbons from Thermosetting Resins. *Carbon* **1970**, *8*, 353-364.
(5) Silverstein, R. M.; Webster, F. X.; J., K. D. *Spectrometric Identification of Organic Compounds*; John Wiley & Sons, Inc., 2005.
(6) Bellamy, L. J. *The Infra-red Spectra of Complex Molecules*; Chapman and Hall, 1975.
(7) Shindo, A.; Izumino, K. Structural Variation During Pyrolysis of Furfuryl Alcohol and Furfural-Furfuryl Alcohol Resins. *Carbon* **1994**, *32* (7), 1233-1243.
(8) Wang, F.; Polavarapu, P. L. Conformational Stability of (+)-Epichlorohydrin. *J. Phys. Chem. A* **2000**, *104*, 6189-6196.
(9) Kalasinsky, V. F.; Wurrey, C. J. Vibrational Spectra and Conformations of (Chloromethyl)cyclopropane and Epichlorohydrin. *J. Raman Spec.* **1980**, *9* (5), 315-323.
(10) Smith, B. C. Organic Nitrogen Compounds V: Amine Salts. In *Spectroscopy*, 2019; Vol. 34, pp 30-37.
(11) Partansky, A. M. A Study of Accelerators for Epoxy-Amine Condensation Reaction. In *Epoxy Resins*, Advances in Chemistry, Vol. 92; American Chemical Society, 1970; pp 29-47.
(12) Savage, G. Thermosetting Resin Matrix Precursors. In *Carbon-Carbon Composites*, Springer Dordrecht, 1993; pp 117-156.
